# Lab to codes: multimodel advanced AI-driven pipelines to drive antimicrobial peptide design for the post-antibiotic era

**DOI:** 10.3389/fcimb.2026.1894311

**Published:** 2026-09-29

**Authors:** Naveed Saleem, Emad El-Omar, Naresh Kumar, Mark Willcox, Xiao-Tao Jiang

**Affiliations:** 1 Microbiome Research Centre, School of Clinical Medicine, Faculty of Medicine & Health, University of New South Wales (UNSW), Sydney, NSW, Australia; 2 School of Chemistry, University of New South Wales (UNSW), Sydney, NSW, Australia; 3 School of Optometry and Vision Science, University of New South Wales (UNSW), Sydney, NSW, Australia

**Keywords:** antimicrobial peptides (AMPs), artificial intelligence (AI), deep learning (DL), generative-AI, machine learning (ML), molecular docking (MD), multi-drug resistance (MDR)

## Abstract

The rapid emergence of global antimicrobial resistance (AMR) has outpaced the development of new antimicrobial agents, necessitating transformative approaches such as AI-driven *in-silico* drug discovery. Antimicrobial peptides (AMPs) are promising alternatives due to their broad-spectrum bactericidal activity and limited susceptibility to resistance. However, the transition from bench to bedside remains constrained by certain challenges, such as instability, potency, and cytotoxicity. Here, well-integrated, multi-model AI-driven pipeline strategies for the mining and discovery of novel AMPs are designed to address these concerns through simultaneous multi-purpose optimization. The proposed architectural frameworks combine discriminative AMP classifiers, quantitative potency, and cytotoxicity screening filters to prioritize AMPs with good efficacy and safety profiles. To ensure novelty, the pipeline integrates multi-layer sequential and genomic screening by adopting alignment and profile-based approaches. Structural refinements are achieved through advanced molecular docking (MD) and protein folding approaches, providing mechanistic insights regarding peptide–target interactions. In parallel, enzymatic susceptibility and stability prediction models are incorporated to optimize AMP pharmacokinetic potentials. Notably, the multi-objective pipeline operates within iterative optimization loops; discriminative and generative AI models and sequential redesign strategies refine candidates based on multi-purpose closed feedback loops across stability, novelty, efficacy, and toxicity outcomes. These systematic and integrated approaches overcome key bottlenecks associated with traditional linear drug discovery, potentially reducing late-stage attrition and accelerating the transformation from *in-silico* predictions to wet-lab validation. Collectively, this review provides reproducible and generalizable blueprints for next-generation antimicrobial agents, demonstrating the computational potentials of AI-driven, multi-model architectural frameworks to tackle the global AMR crisis and favour precision design of AMPs with better therapeutic indices.

## 1 Introduction

In the 21^st^ century, antimicrobial resistance (AMR) has evolved as one of the most serious global health concerns, undermining decades of progress in modern medicine (Selvaraj et al., 2025; Jesudason, 2026). Recent global AMR trends have indicated that millions of deaths are directly attributable to bacterial AMR annually and continue to rise across both low-and high-income countries (Selvaraj et al., 2025; Jesudason, 2026). The World Health Organization (WHO) highlights that these trends are increasing exponentially across critical priority pathogens, thereby compromising the effectiveness of last-resort antimicrobial therapies and jeopardizing clinical practices such as chemotherapy and regular surgical and transplantation procedures (Selvaraj et al., 2025; Jesudason, 2026). Without transformative interventional approaches, AMR is going to impose serious health and economic burdens, potentially exceeding morbidity, and mortality outcomes within the coming decades (Sadeeq et al., 2025).

Despite these escalating threats, traditional antibiotic development has remained critically fragile (Palmo et al., 2026). At present, few antimicrobial candidates are in clinical development, and few of these use novel mechanisms of action (Palmo et al., 2026) (**Figure 1** and **2**). Most current antibiotic discovery paradigms are dependent on incremental structural modifications, vigorous empirical screening, and testing. These often fail to explore the vast chemical and biological spaces that may be open for new development, which are essential to identify next-generation candidates due to high costs (Panjla et al., 2024; Chen et al., 2025) (**Figure 2**). Consequently, there is an urgent need to transition from traditional discovery. This is where integrative and data-oriented approaches can help to accelerate drug innovation strategies (Panjla et al., 2024; Chen et al., 2025).

**Figure 1:**
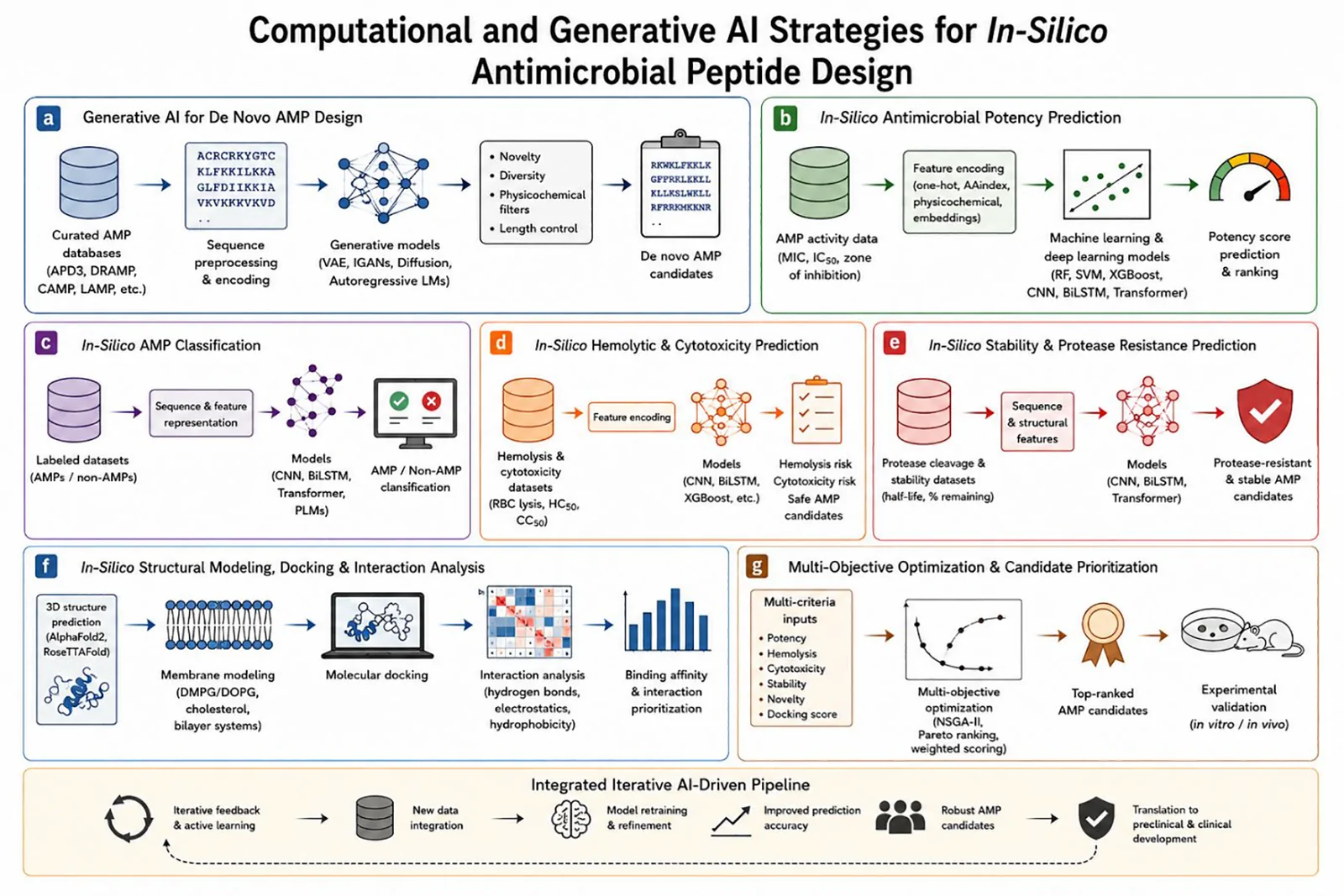
The graphical abstract demonstrates an integrated multi-model AI-driven strategy. **(a–e)** In-silico frameworks can be used for the mining and optimization of novel AMPs through iterative and sequential integration of computational approaches. **(a, c)** Generative-AI enables *de novo* AMP design from diverse datasets, while discriminative AMP classifiers predict AMPs. **(b)** MIC-regression models to prioritize AMPs with higher bactericidal activity. **(d)** Haemolysis and cytotoxicity prediction models serve as early safety filters, whereas novelty screening using genome-level tools identifies previously uncharacterised AMPs. **(e)** In parallel, stability screening assesses protease susceptibility. **(f)** Molecular docking provides mechanistic insights about peptide–target interactions and guides structure-based optimization. **(a–g)** All the components are integrated within feedback-driven optimization loops, ensuring multi-purpose refinements across safety, novelty, potency, and stability. Collectively, this approach provides a generalizable methodology to accelerate the clinical translation of novel AMPs in the era of AI-driven computational biology. The image was created with BioRender (https://www.biorender.com).

**Figure 2:**
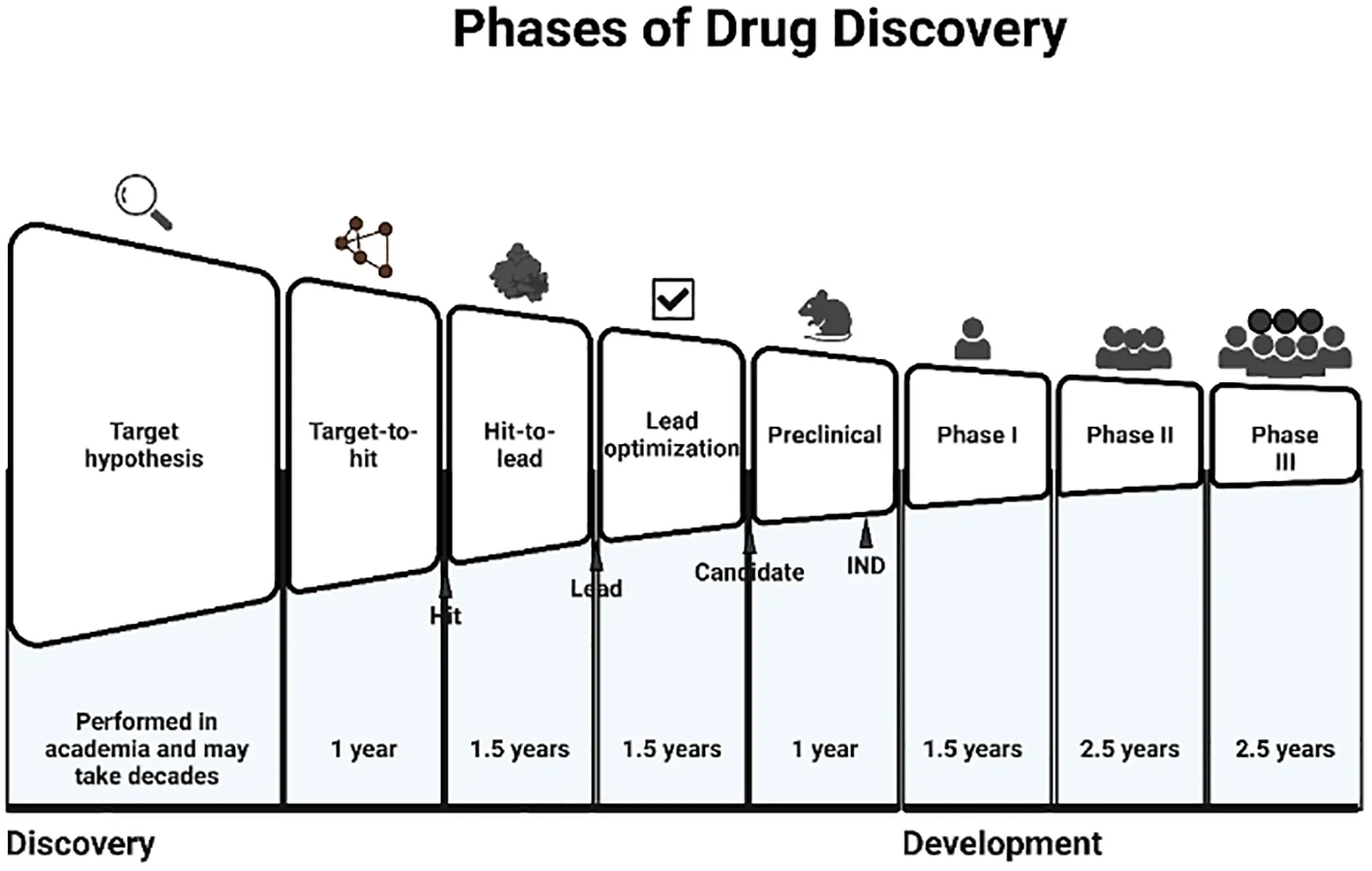
Schematic representation of different phases and timeline for drug discovery, i.e., target identification, lead discovery and optimization, preclinical and clinical evaluation to assess efficacy and safety, guiding the progression of novel candidates towards translation from bench to bedside. The image was created with BioRender (https://www.biorender.com).

Artificial intelligence (AI) has emerged as a transformative paradigm for antibiotic discovery, enabling the systematic interrogation of available large-scale chemical and biological datasets (Musin and Asyanova, 2025). AI-driven approaches, such as machine learning (ML) and deep learning (DL), generative modelling, and large language models (LLMs), have shown the capabilities to mine and identify novel candidates, predict their physicochemical properties, and optimize molecular designs with better efficacy and safety profiles (Zervou et al., 2024; Li et al., 2025; Mittal et al., 2025). Notably, studies have leveraged LLM architectural frameworks to construct end-to-end pipelines capable of generating new antibiotics from vast sequential spaces, achieving higher potency and limited cytotoxicity during wet-lab validation (Chen et al., 2025; Saleem et al., 2025). These advancements have underscored the paradigm shifts from linear and hypothesis-driven drug discovery toward iterative and AI-oriented designs and build–test cycles (Chen et al., 2025; Saleem et al., 2025).

Amongst various emerging modalities, antimicrobial peptides (AMPs) represent one of the most promising candidates, having rapid bactericidal mechanisms and broad-spectrum activity with limited propensity for developing resistance relative to conventional antimicrobial agents (Oliveira Júnior et al., 2025; Torres et al., 2025; Veldsman et al., 2025). However, their clinical translation remains limited by challenges such as suboptimal pharmacokinetics, proteolytic instability, and cytotoxicity. Addressing these limitations requires holistic frameworks, integrating multi-dimensional predictive and generative modelling alongside structural and functional optimization (Perišić et al., 2025; Sun et al., 2025; Szymczak et al., 2025).

The current review proposes multi-model AI-driven strategies for AMP mining and optimization, designed to bridge the gap between *in-silico* prediction and translational applicability (**Figure 3**). This multifaceted framework integrates advanced discriminative AMP classifiers for activity prediction and generative models for *de novo* AMP design and generation (Noor et al., 2025; Saleem et al., 2025; Sun, 2025; Szymczak et al., 2025) (**Figure 3**); *in-silico* potency filters to predict antimicrobial efficacy against proposed pathogens (Noor et al., 2025; Su et al., 2025; Sun et al., 2025) (**Figure 3**); haemolysis and cytotoxicity predicted modules to ensure host safety (Noor et al., 2025; Su et al., 2025; Sun et al., 2025) (**Figure 3**); novelty models to enhance chemical and structural diversity, both at the genomic and protein sequence levels (National Center for Biotechnology I., 2026) (**Figure 3**); structural alignments and folding predictions to limit three-dimensional conformations (**Figure 3**); proteolytic-resistant mappings to optimize peptide stability and *in-vivo* persistence (**Figure 3**).

**Figure 3:**
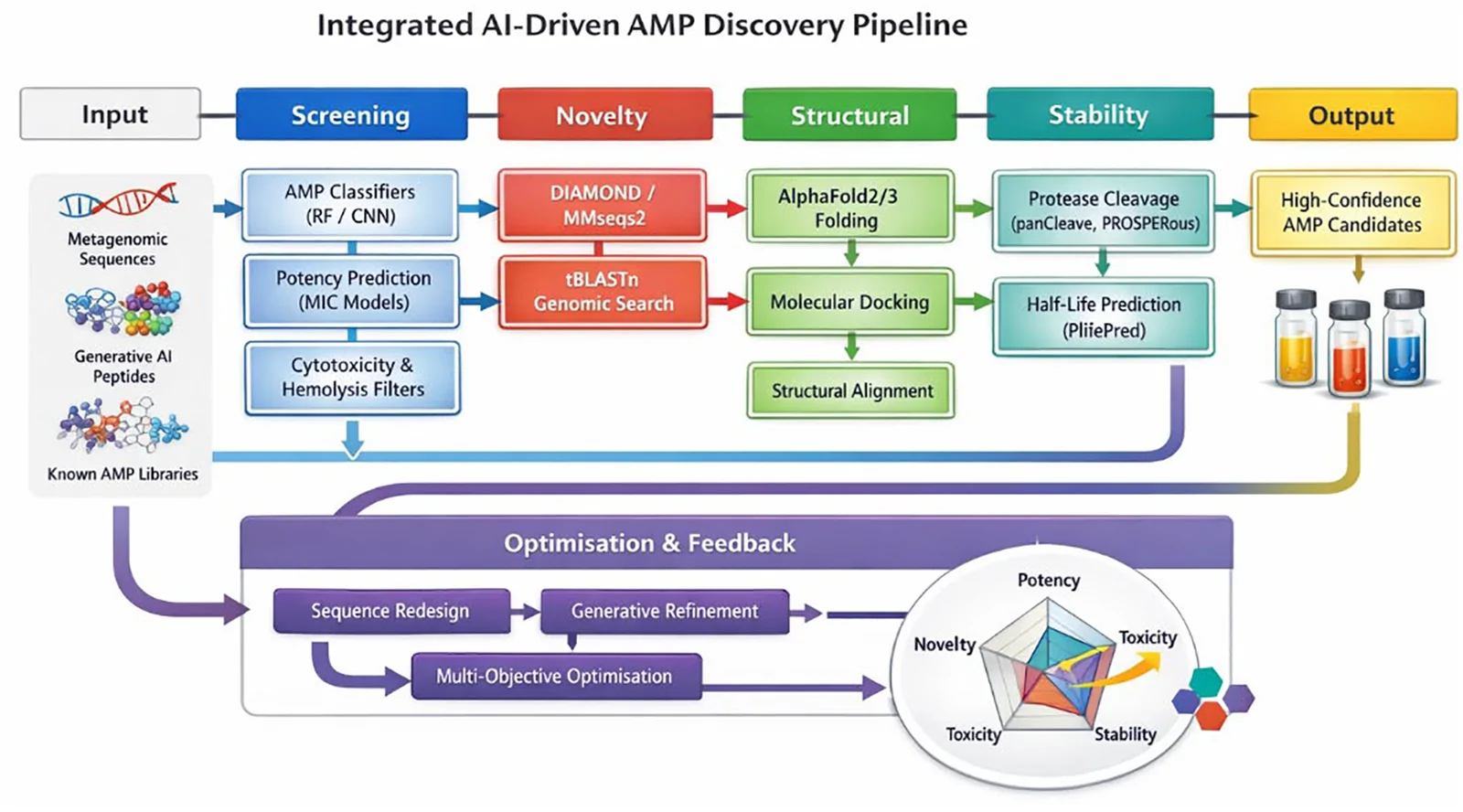
A well-integrated multi-model AI-driven strategy can mine and optimize novel AMPs using discriminative AMP classifiers, followed by MIC-regression models to prioritize AMPs with better efficacy. Haemolysis and cytotoxicity prediction models serve as initial safety filters, whereas novelty filters using genome-level tools identify previously undiscovered AMPs. Molecular docking provides mechanistic insights about peptide–target interactions, and stability screening assesses protease susceptibility. Altogether, this generalizable methodology will speed up the clinical translation of novel AMPs to combat the global AMR crisis. The image was created with BioRender (https://www.biorender.com) & assisted by ChatGPT (GPT-5.5) for scientific visualization.

The incorporation of different AI-driven architectural frameworks within a unified pipeline strategy optimizes multi-purpose outcomes across novelty, stability, efficacy, and safety, addressing key bottlenecks which historically limit AMP’s clinical translation (Zhao et al., 2025; Palmo et al., 2026) (**Figure 3**). Importantly, the integration of structural, functional, and proteolytic constraints at initial stages demonstrates critical advancements over pre-existing pipelines, which prioritize activity-based predictions in isolation only (Musin and Asyanova, 2025; Wang et al., 2025d).

This review highlights recent progress in AI-enabled AMP discovery and contextualizes the adoption of multi-model strategies within the broader landscape for developing next-generation novel therapeutics. Such integrative frameworks are critical for navigating the complexities associated with AMP designs and for accelerating the clinical translation of computational discoveries to combat drug-resistant pathogens (Veldsman et al., 2025; Palmo et al., 2026). This review proposes the adaptation of multi-model advanced AI-driven approaches with context for novel AMP discovery, tailored for computational biologists, microbiologists, and clinicians, showing interdisciplinary translational aspects. By doing so, this highlights that the future goals must be directed towards precision-driven antimicrobial development, which will be capable of addressing the global AMR crisis.

### 1.1 Instability and non-selectivity as pre-design constraints, not post-screening failures

Traditional antibiotic discovery paradigms, which progress linearly from drug design and synthesis to *in-vitro* and *in-vivo* validation, may have historically undermined antimicrobial development (Blaskovich and Cooper, 2025) (**Figure 4**). Whilst these conventional frameworks have enabled the discovery of several first-generation antibiotics, they are increasingly unable to address the many new challenges (Blaskovich and Cooper, 2025; Farha et al., 2025). One of the key limitations is their inherently low throughputs, where the majority of compounds fail at later validation stages due to poor efficacy and drug-related cytotoxic events (Blaskovich and Cooper, 2025; Farha et al., 2025). Such iterative approaches usually require extensive financial resources and experimental efforts alongside substantial time, often extending across a decade from bench to bedside (Farha et al., 2025) (**Figure 4**). Consequently, these pipelines are economically unsustainable, and the probability of success remains low; therefore, investments for antibiotics are comparatively limited both from the public and private sectors (Farha et al., 2025; Manan et al., 2025) (**Figure 4**).

**Figure 4:**
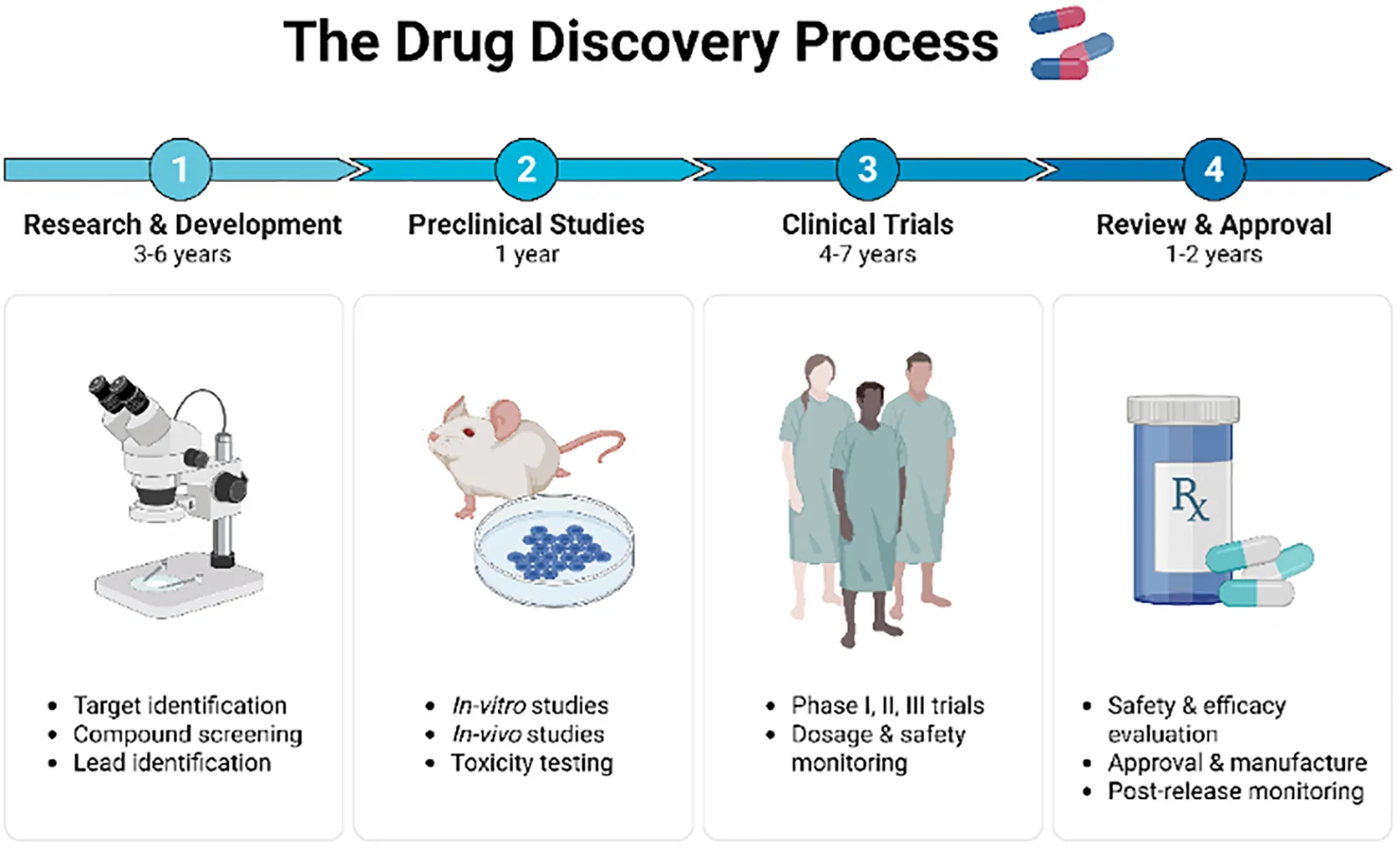
Overview of drug development: a multi-staged process, starting from research (target identification and optimization), preclinical and clinical evaluation, and regulatory reviews for final approval. Each step involves rigorous assessments for pharmacological, efficacy, and safety profiles, translating the promising candidates from bench to bedside. The image was created with BioRender (https://www.biorender.com).

Recently, the WHO warned of the paucity of antibiotics currently in clinical development, and the low numbers of these representing truly novel therapeutic agents (Daruka et al., 2025). Mostly, candidates are derived from pre-existing scaffolds, thereby offering accretive improvements over transformative outcomes (Daruka et al., 2025; Gargate et al., 2025). This stagnation is compounded by technical bottlenecks; the difficulty of identifying compounds that penetrate bacterial membranes, evade efflux pumps, and retain bactericidal activities against drug-resistant pathogens (Daruka et al., 2025; Saleem et al., 2026). Traditional pipelines have failed to tackle the rapid evolutionary resistance approaches, creating widening gaps between therapeutic availability and clinical needs (Daruka et al., 2025; Gargate et al., 2025).

The sequential nature of the conventional workflows has introduced a critical structural inefficiency: decision-making is suspended until later-stage wet-lab validation. Instability, non-selectivity, and cytotoxicity issues are usually detected during drug synthesis and biological testing, leading to candidate elimination due to cost attrition (Gargate et al., 2025). These “fail-late” paradigms are particularly problematic for peptide-based therapeutics, as protease instability and host cytotoxic events are context-dependent and challenging to streamline through pragmatic iterations (Oliveira Júnior et al., 2025). Altogether, these hindrances underpin fundamental bottlenecks associated with antibiotic development, necessitating a shift toward predicted and multi-purpose design frameworks which are capable of addressing stability, efficacy, and safety simultaneously at the initial stages of drug discovery (Manan et al., 2025).

### 1.2 AI, as the first stage of stability, efficacy, and safety validation

#### 1.2.1 Discriminative AMP classifiers to predict AMP mining and discovery

Discriminative ML models are considered the computational backbone for early AMP discovery, enabling them to mine and classify candidates based on their likelihood of demonstrating adequate bactericidal activities (Meng and Proteins, 2025; Musin and Asyanova, 2025) (**Figure 5**). Classical ML methods, such as Support Vector Machines (SVM), Random Forests (RF), k-Nearest Neighbours (kNN), and gradient-boosting frameworks, leverage engineered characteristics based on parameters, i.e., amino acid composition, physicochemical properties, and evolutionary descriptors, to differentiate AMPs from non-functional candidates (Meng and Proteins, 2025; Musin and Asyanova, 2025) (**Table 1**). These approaches are compatible with high-dimensional biological datasets for capturing non-linear relationships between sequential features and antibacterial properties (Waghu and Idicula-Thomas, 2020; Meng and Proteins, 2025).

**Figure 5:**
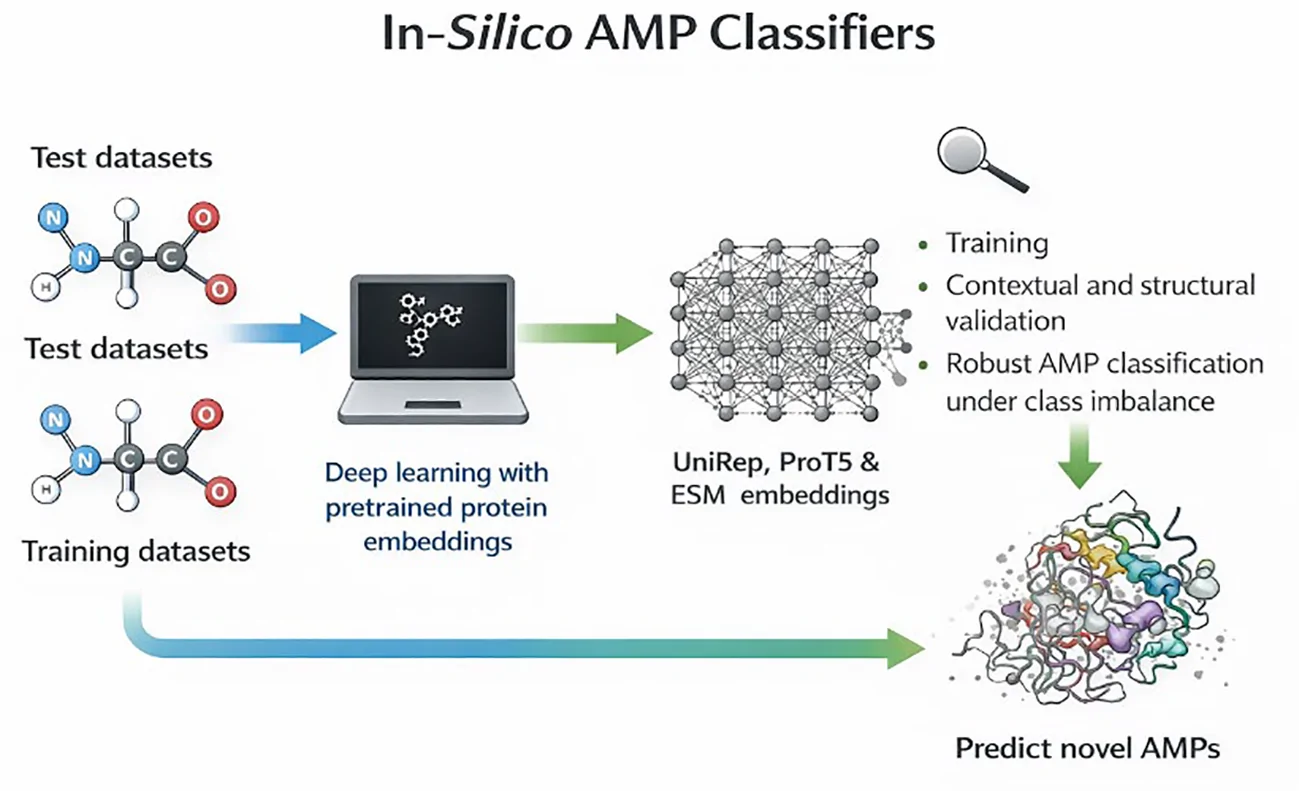
Classical and deep learning–based AMP classifiers with integrated diverse architectural frameworks can be used to mine and predict antimicrobial potentials. These models assign probability scores to reflect AMP likelihoods, enabling systematic ranking of high-confidence AMPs for downstream wet-lab analysis. The image was created with BioRender (https://www.biorender.com).

**Table 1:** Different discriminative AMP classifiers (machine & deep learning approaches) to predict AMP mining and discovery.

Models & year	Architectural framework	Performance metrics	Key features & experimental validations
a. Classical ML (RF, SVM, GBMs)			
AntiBP2 & AntiBP3 2017/2024	SVM	AntiBP2: Accuracy (92.1%), MCC (0.84); AntiBP3: AUC (0.93–0.98), (MCC up to 0.86)	SVM-based predictors used balanced +ve and -ve training datasets using residue contact maps to integrate with in silico toxicity screening. Predicted antimicrobial activity. Experimental Validation: No, computational validation only (Gabere and Noble, 2017; Bajiya et al., 2024).
AmpClass 2024	Ensemble ML (XGBoost, RF, NN, DT)	Accuracy (93.2%)	Classification & regression approaches to predict novel AMPs. Predicted antimicrobial activity. Experimental Validation: No, computational validation only (Mera-Banguero et al., 2024).
AmpGram 2020	N-gram encoding & stacked random forests.	AUROC (0.98)	Predicted longer peptides (>10 A.A), used for high-throughput proteomics-based AMPs. Predicted antimicrobial activity. Experimental Validation: No, computational validation only (Burdukiewicz et al., 2020).
AmPEP 2018	RF classifiers	Accuracy (96%), MCC (0.90), AUC (0.99)	Provided distribution patterns of amino acid features, with higher accuracy, simplicity, & reduction capability. Predicted antimicrobial activity. Experimental Validation: No, computational validation only (Bhadra et al., 2018).
AmPEPpy 2021	Random Forest (RF)	Accuracy (91%)	It predicted plant peptides using amino acid composition features. Experimental Validation: No, computational validation only (Lawrence et al., 2021; Veldsman et al., 2025).
CalcAMP 2023	LightGBM, ensemble& tree algorithms	Accuracy 86–90% (best RF model 90%)	It used a curated dataset of validated short AMPs and classified them by spectrum. Predicted antimicrobial activity. Experimental Validation: Yes, *in-vitro* validation (Bournez et al., 2023).
CAMP-R3/R4 2016/2023	ML classifiers (SVM, RF, DA, HMM)	R3: Accuracy (90.5%), R4: AUROC (0.93)	Multi-model approaches showed improvement in performance, i.e., area under the receiver operating characteristic curve. Predicted antimicrobial activity. Experimental Validation: N/A (Waghu et al., 2014; Waghu et al., 2016; Gabere and Noble, 2017; Gawde et al., 2022; Liu et al., 2022; Musa, 2024).
iAMPpred (AMPredict) 2021	SVM	Accuracy (74–88% depending on class)	It used physicochemical descriptors like hydrophobicity & charge. Predicted antimicrobial activity. Experimental Validation: Unclear (Donaghy et al., 2020; Norvell et al., 2021).
iAMP-2L 2013	2-layered FKNN with PseAAC	Overall accuracy (87.6%)	The multi-model approach predicted five function categories. Predicted antimicrobial activity. Experimental Validation: No, computational validation only (Xiao et al., 2013).
MLAMP (Multi-Label AMP predictor) 2016	Ensemble of different ML, i.e., SMOTE & PseAAC	Micro-F1 (0.78)	Multi-label peptide functional models identified AMPs & their biological roles. Experimental Validation: No, computational validation only (Lin and Xu, 2016).
MLBP 2022	Multi-scale ML alongside CNN & BiGRU	Accuracy (86%)	It processed raw sequential vectors without having LLM inputs. Predicted antimicrobial activity. Experimental Validation: No, computational validation only (Li et al., 2022; Tang et al., 2022).
PEPred-Suite 2019	Different RF models	Accuracy (89%), AUC (0.92)	Using sequence-based descriptors & adaptive learning to predict efficacy & safety profiles. Experimental Validation: No, computational validation only (Wei et al., 2019).
Target-AMP 2022	RF, SVM, KNNs	Accuracy (93.8%)	Used evolutionary data & composition features with multiple classifiers to predict AMP. Predicted antimicrobial activity. Experimental Validation: No, computational validation only (Jan et al., 2022).
b. Deep learning (CNN, GNN, RNN, Transformers, others)			
AI4AMP 2021	CNN, LSTM, DNNs	Accuracy (91.7%)	Used PC6 & autocovariance to predict novel AMPs. Predicted antimicrobial activity. Experimental Validation: No, computational validation only (Lin et al., 2021).
AMPlify 2022	Bi-LSTM, Multi-head attention using Word2Vec tokens	AUROC (0.984), AUPRC (0.986). F1 (0.94)	An attention-based model to validate against the WHO priority pathogens & used ensemble learning to improve robustness. Experimental Validation: Yes, *in-vitro* validation (Li et al., 2022; Li et al., 2023).
AMPpred-CNN 2023	1D CNNs	Accuracy (92%)	Peptide sequences were encoded using CTD descriptors to predict AMPs. Predicted antimicrobial activity. Experimental Validation: No, computational validation only (Mitra and Singh, 2023).
AMP Scanner AMP Scanner v2 2018	CNN, RNN, deep neural network	Accuracy (92%, MCC (0.85)	Predicted efficacy based on physicochemical properties. Predicted antimicrobial activity. Experimental Validation: No, computational validation only (Veltri et al., 2018; Musin and Asyanova, 2025).
APIN 2019	CNN with embedded layers	Accuracy (94%)	Predicted AMPs using convolutional architectures directly from sequences. Predicted antimicrobial activity. Experimental Validation: No, computational validation only (Su et al., 2019).
CG-AMP 2025	ProtT5 pre-trained protein LM embeddings. Supervised contrastive learning. Gated-CNN with Gated Linear Units (GLU) for AMP classification	AMPlify benchmark: Accuracy (0.9497) F1 (0.9508). DAMP benchmark: Accuracy (0.9403) F1-score (0.9392)	Discriminative AMP representations using contrastive learning, integrated sequence semantics, evolutionary information and physicochemical properties for classification. Experimental Validation: No, computational validation only (Li et al., 2025).
deepAMPNet 2024	Pretrained Bi-LSTM, GNNs, & structural graphs	AUROC (0.97)	Alongside structure and language-derived encodings, it predicted delivering accuracy and biological insights. Predicted antimicrobial activity. Experimental Validation: No, computational validation only (Zhao et al., 2024).
Deep-AmPEP30 2020	CNN trained on PseKRAAC for shorter peptides	Accuracy (92.6%)	Optimized & predicted shorter peptides. Experimental Validation: No, computational validation only (Yan et al., 2020; Lobanov et al., 2024; Nguyen et al., 2024).
DMAMP 2024	CNN, Residual CNN Blocks, PSSM	Accuracy (91.3%)	A multi-task predictive model used CNN–residual architecture & evolutionary features fusion for robust and accurate prediction. Predicted antimicrobial activity. Experimental Validation: No, computational validation only (Meng et al., 2024).
GAC-BiTCNN-AMP 2026	Hybrid GANs, capsule networks, bidirectional Temporal CNN alongside UniRep, ProtTrans-T5, ESM-2 and PsePSSM-DCT	Accuracy (97.42%) F1 (97.83%) AUC (99.54%) MCC (0.923)	Predicted AMPs with latent-feature analysis. Used as a discriminative & generative-augmentation hybrid model. Experimental Validation: No, computational validation only (Ali et al., 2026).
HDM-AMP 2021	ESM-1b, Deep Forest	Accuracy (89.5%)	It was interpreted and predicted using an ensemble of decision trees. Experimental Validation: No, computational validation only (Yu et al., 2021).
iAMPCN 2023	CNN	Accuracy (93.4%)	It used handcrafted features & classifies using a CNN without LLM embeddings. Predicted antimicrobial activity. Experimental Validation: No, computational validation only (Xu et al., 2023; Han et al., 2025).
iAMP-CA2L	CNN, Bi-LSTM, & SVM	Accuracy (91.2%)	It used hybrid SVM models for final classification with a dual-task focus. Experimental Validation: No, computational validation only (Xiao et al., 2021; Ruffolo and Madani, 2024).
LMPred 2021	ProtTrans, CNN classifiers, T5, & XLNet	Accuracy (92–93%)	Bridged protein sequence understanding & learned pattern detection. Predicted antimicrobial activity. Experimental Validation: No, computational validation only (Dee, 2022).
MBC-attention 2023	CNN, Attention, ML	MCC (0.81)	It focused on critical residual motifs causing membrane disruption using attention models. Predicted antimicrobial activity. Experimental Validation: No, computational validation only (Yan et al., 2023; Dong et al., 2025a).
sAMPpred-GAT 2022	Graph Neural Network (GAT) & ML	AUROC (0.95)	Graph attention networks leverage sequence-to-graph conversion. Experimental Validation: No, computational validation only (Lv et al., 2022).
SGAC 2026	Spatial GNN-based AMP classifiers used Cα-based peptide graphs	F1 (94.83 0.77) MCC (93.02 0.56)	Predicted AMPs from peptide sequences alongside inferred/predicted 3D structural information. Highly relevant for metagenomic AMP mining. Experimental Validation: No, computational validation only (Wang et al., 2026).
TP-LMMSG 2024	Deep learning GNN on proteins, LM residues	Accuracy (94%)	Protein-LM, based on each residue, provided multi-scaled structural information. Predicted antimicrobial activity. Experimental Validation: No, computational validation only (Chen et al., 2024).
c. Ensemble models with hybrid frameworks			
AMP-BRET 2023	RF, ProtBERT Transformer with fine-tuning for regression	Accuracy (92.1%)	Demonstrated high precision & transfer learning from protein corpora. Predicted antimicrobial activity. Experimental Validation: Unclear (Lee et al., 2023).
AMPpred-EL 2022	Ensemble ML (Logistic Regression & LightGBM)	Accuracy (93.8%)	It combined multiple ML components for stronger AMPs prediction. Predicted antimicrobial activity. Experimental Validation: No, computational validation only (Lv et al., 2022).
AMPpredMFA 2024	LSTM, CNN, Attention, & MLP	AUROC (0.97)	It integrated local (CNN) and long-range (LSTM) sequence features to predict features. Experimental Validation: No, computational validation only (Li et al., 2024).
AMP-META 2024	Light GBM (LGBM) comprises different AMP tools	Accuracy (95%)	It predicted physicochemical descriptors using larger datasets. Predicted antimicrobial activity. Experimental Validation: Yes, *in-vitro* validation (Jorge et al., 2012; Tsai et al., 2024).
E-CLEAP 2024	Ensemble of four MLP classifiers	Accuracy (97.3%) (AAC features), Accuracy 84.0% (PseAAC features), F1 (0.93)	Ensemble neural classifiers performed high-accuracy AMP classification. Predicted antimicrobial activity. Experimental Validation: No, computational validation only (Wang, 2024).
d. Transformers-based models			
AMPTrans 2023	LSTM, Transformer, RF, SVM with adaptive QSAR	Accuracy (93%)	QSAR-enabled designer facilitated sequence guidance & novelty. Experimental Validation: No, computational validation only (Mao et al., 2023).
AMP-ProtBERT 2025	Fine-tuned ProtBERT	AUROC (0.98)5 (ProtBERT AMP classifier), Accuracy (94%)	iAMP-bert (ESM-2) pretrained models outperformed for AMPs prediction and antimicrobial activity. Experimental Validation: No, computational validation only (Lee et al., 2023; Abbasi et al., 2025; Georgoulis et al., 2025).
e. Specialized supportive models			
ESKAPEE-MICpred 2024	LSTM, CNN, & MLP	R² = 0.82(Species-specific MIC regression model	It used sequence-derived descriptors to predict activity. Experimental Validation: No, computational validation only (Sharma et al., 2024).
EnDL-HemoLyt 2024	LSTM, CNN, & MLP	AUROC (0.97), MCC (0.80)	It optimized the therapeutic index by predicting haemolysis. Experimental Validation: No, computational validation only (Maasch et al., 2023).
StaBle-ABPpred 2022	BiLSTM	AUROC (0.95); Accuracy (0.91), MCC (0.82), AUPRC (0.97)	It predicted peptides’ stability and activity using Word2Vec embeddings. Experimental Validation: No, computational validation only (Singh et al., 2022).
SMEP/SAMP 2023/2024	LSTM, XGBoost	Accuracy (90%), F1 (0.89)	Used different libraries such as nonapeptide, heptapeptide, and octapeptide to predict efficacy & safety. Experimental Validation: No, computational validation only (Huang et al., 2023; Feng et al., 2024; Panjla et al., 2024).
f. Data-centric ML			
GMSC-mapper 2024	Modified version of Prodigal, i.e., RF	Accuracy (89%)	It identified and annotated smaller proteins using microbial (meta)genomes. Experimental Validation: No, computational validation only (Duan et al., 2024; Neville and Lawley, 2025; Szymczak et al., 2025).
Macrel 2025	RF with metagenomics mining, LP models	AUROC (0.97–0.99 depending on datasets)	It predicted sequence-derived descriptors using proteome, genome, & transcriptome to show efficacy & safety. Experimental Validation: Yes, validation reported for predicted AMPs (Gonzalez-de la Rosa et al., 2025; Wu et al., 2025).
g. Emerging automated ML models			
AutoPeptideML 2024	AutoML-based peptide classifier with evolutionary features	Accuracy (90%)	It automated feature engineering from evolutionary indices to predict antimicrobial activity. Experimental Validation: No, computational validation only (Fernández-Díaz et al., 2024).
APEX 2024	RNN, ATT, & MLP	Accuracy (92%)	Deep learning aided by Molecular de-extinction to show *in-vitro* & *in-vivo* properties. Experimental Validation: Yes, *in-vitro* & *in-vivo* validation (Wan et al., 2024).
PrMFTP 2022	Multiscale CNN, BiLSTM, Multiheaded self-attention approach	Accuracy (93%), F1 (0.92)	It combined architectural models, i.e., CNN, BiLSTM, and self-attention, which were not derived from LLM. Experimental Validation: No, computational validation only (Yan et al., 2022).
h. Other ML/LLM models			
AMPActiPred 2024	Multi-class ML functional	Accuracy (91%)	A three-stage framework employed peptide descriptors to capture compositional and physicochemical properties & activity. Experimental Validation: No, computational validation only (Yao et al., 2024).
AMP-Distillation 2026	Knowledge-distillation frameworks for AMP prediction	NA	AMP classification for high-throughput screening. Experimental Validation: No, computational validation only (Khorramfard et al., 2026).
Ansari & White 2023/2024	RNN, LSTM	AUROC (0.93)	Semi-supervised method predicted peptide efficacy 7 safety via positive-unlabelled learning (PU). Experimental Validation: No, computational validation only (Ansari and White, 2023; Ansari and White, 2024).
Biochemical-driven ML pipeline 2026	RF classifier alongside a biochemical-knowledge-based ML for AMP screening	NA	Predicted potent AMPs using biochemical constraints. 18.5% of generated AMPs showed antimicrobial activity (MIC ≤10 μg/mL), and 38.9% had MIC ≤50 μg/mL. Experimental Validation: Yes, *in-vitro* validation only (Yang et al., 2026).
Capecchi et al. 2021	RNN, GRU, SVM; MLP	AUC (0.95)	ML used DBAASP to predict short non-haemolytic & microbial activity of AMPs. Experimental Validation: Yes, *in-vitro* validation (Capecchi et al., 2021).
PepGraphormer 2026	ESM-2, Graph Attention Network (GAT)	Matched state-of-the-art AMP predictors across seven benchmark datasets	Predicted AMP activity without requiring 3D structures & combined protein LLMs semantics with graph relational learning. Experimental Validation: No, computational validation (Lin et al., 2026).
Zhuang et al. 2024	QSVM (Quantum Support Vector Machine)	Accuracy (94%) data-dependent	It predicted sequence-derived descriptors to predict safety profiles. Experimental Validation: No, computational validation (Zhuang et al., 2024).

Experimental validation referred to wet-lab testing of the model-predicted model. Computational cross-validation and retrospective database benchmarking were not considered experimental validation. “Unclear” indicated that experimental confirmation could not be established unequivocally from the cited study; N/A denoted databases or computational resources for which experimental validation is not directly applicable.

Apart from binary AMP classification, discriminative models have provided support for high-throughput screening of diverse datasets, i.e., metagenomics, proteomics, and other commercially available datasets (Meng and Proteins, 2025; Musin and Asyanova, 2025). Architectural Frameworks, i.e., RF -based AmPEP and gradient-boosting architectures (e.g., XGBoost, LightGBM), have prioritized novel AMPs, subsequently experimentally validated against clinically relevant pathogens (*Escherichia coli* and *Staphylococcus aureus*) (Bhadra et al., 2018; Mera-Banguero et al., 2024). Importantly, ensemble frameworks have integrated multiple AMP classifiers to limit individual model bias and have improved generalization by achieving predictive accuracies around 95% (Mera-Banguero et al., 2024; Zhong et al., 2024). Altogether, these strategies illustrate the critical role of discriminative models for narrowing vast sequential spaces into experimentally tractable candidates for clinical translation (Mera-Banguero et al., 2024; Zhong et al., 2024).

Recent studies have highlighted the scope of discriminative modelling by integrating DL architectures, i.e., convolutional neural networks (CNNs), recurrent neural networks (RNNs), and long short-term memory (LSTM) frameworks (Zhao et al., 2024; Lin et al., 2025). These models have captured local motifs and longer-range dependencies by analysing sequences as analogous to linguistic inputs, with improved representations for sequence–function relationships (Zhao et al., 2024; Lin et al., 2025). Deep classifiers, which are trained on larger AMP databases, have shown better performance in comparison to conventional feature-oriented models, having accuracies exceeding 90% and stronger correlations with *in-vitro* validated antimicrobial activities (Ramazi et al., 2022; Zhang et al., 2023). Hybrid architectural frameworks combining physicochemical features alongside sequential embeddings have upgraded predicted robustness, especially supported by transfer learning outputs from large protein corpora (Wang, 2024; Wang et al., 2025c).

Transformer models represent greater evolutionary changes for discriminative AMP predictions, leveraging attention mechanistic approaches to capture global sequential contexts and complex residue–residue interactions (Rives et al., 2021; Lee et al., 2023; Wang et al., 2025c). Fine-tuned protein-based LLMs, BERT-derived architectures, have given better outcomes for both AMP classification and regression tasks by predicting MICs (minimum inhibitory concentrations) against resistant pathogens (Stokes et al., 2020; Rives et al., 2021; Ma et al., 2022). Collectively, these models have reduced dependency on handcrafted features by learning directly from raw sequential datasets, enabling screening of datasets containing millions of candidates (Stokes et al., 2020; Rives et al., 2021; Ma et al., 2022). However, their performances remain contingent on the availability of larger, diverse training datasets with careful validations to mitigate bias and overfitting (Stokes et al., 2020; Rives et al., 2021; Ma et al., 2022).

Despite having higher predictive accuracy, discriminative AMP classifiers have key limitations for translation (Stokes et al., 2020). Most models are trained on overlapping benchmark datasets, limiting their generalizability (Stokes et al., 2020). Furthermore, classification accuracy is not sufficient to capture clinical endpoints, such as stability, potency, and toxicity (Noor et al., 2025; Szymczak et al., 2025; Zhang et al., 2025). Consequently, discriminative models are considered essential for AMP discovery and rapid filtering to prioritize candidates, but they must be incorporated alongside multi-purpose, AI-driven pipelines for downstream predictions of pharmacological stability, efficacy, and safety profiles (Noor et al., 2025; Szymczak et al., 2025; Zhang et al., 2025). This multifaceted integration can mark necessary progressions from isolated predictive models toward holistic and AI-based frameworks for AMP design and prediction (Noor et al., 2025; Szymczak et al., 2025; Zhang et al., 2025). Despite reported higher classification metrics, direct comparison amongst discriminative models remained constrained by differences in dataset provenance, validation strategies and negative-set construction; these limitations are critically discussed in section 1.3.3.

#### 1.2.2 Generative AI models for *de novo* AMP design

Discriminative models are used to score and prioritize pre-existing peptide sequences, while generative frameworks demonstrate fundamental shifts toward *de novo* design and generation of entirely new AMPs by learning underlying sequential distributions (Lin et al., 2023; Wang et al., 2024a; Zervou et al., 2024). Deep generative architectural frameworks, such as diffusion models, generative adversarial networks (GANs), large language models (LLMs), and variational autoencoders (VAEs), have enabled exploration of previously inaccessible and vast sequential spaces (Van Oort et al., 2021; Lin et al., 2023; Zervou et al., 2024; Guan et al., 2025) (**Figure 6**, **Table 2**). These models are highly effective for addressing the traditional discovery pipelines’ limitations by incorporating multi-purpose outputs, such as stability, potency, and limited toxicity, directly within the design processes.

**Figure 6:**
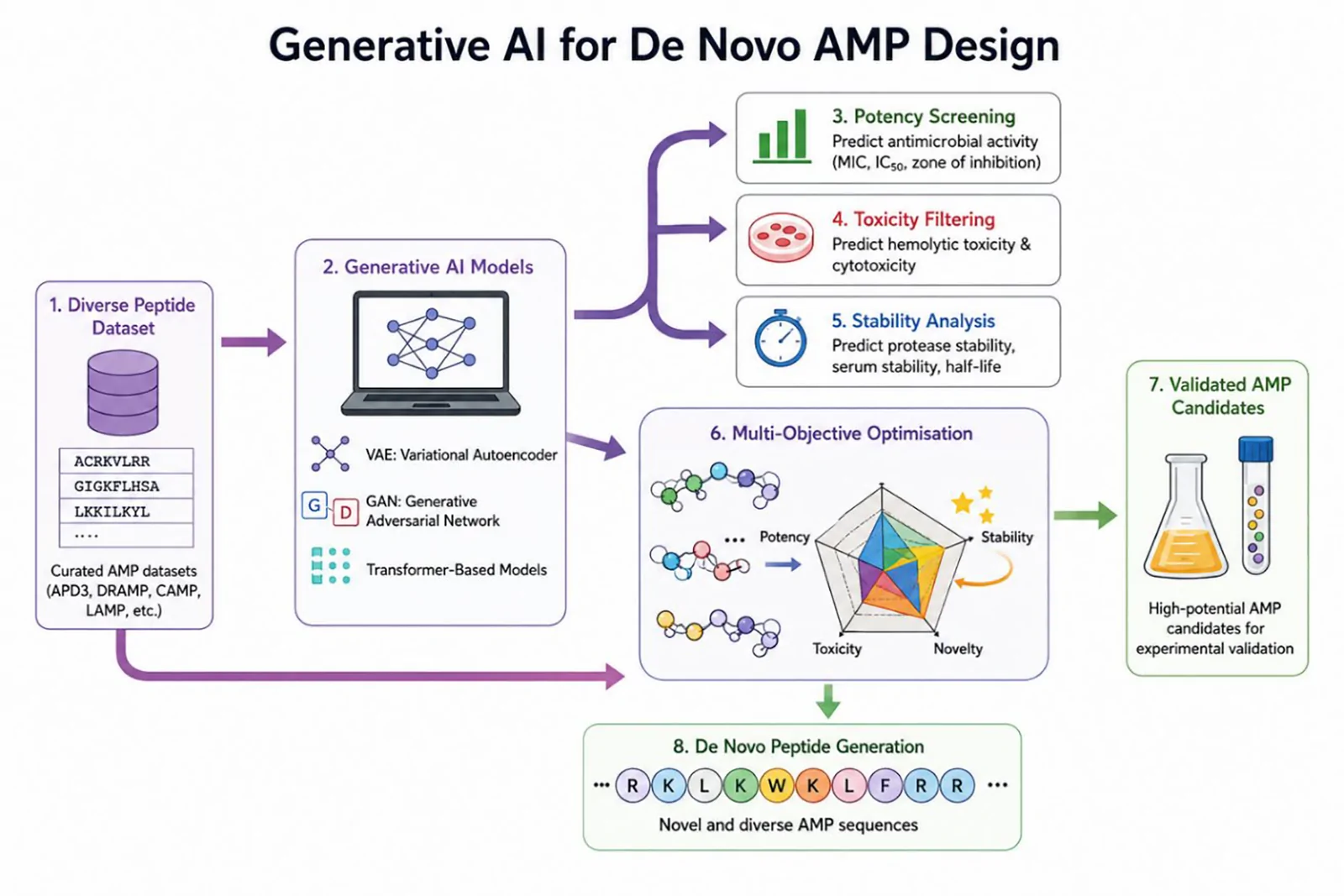
(1–8) Multi-purpose generative AI-driven pipelines can be used for *de novo* design, optimization, and generation of novel AMPs. It depends on robust, functional scoring to predict and optimize novel AMP candidates beyond natural diversity. The image was created with BioRender (https://www.biorender.com).

**Table 2:** Generative-AI models for *de novo* design and generation of novel AMPs.

Generative methods for AMPs discovery			
Models & year	Architectural frameworks	Control generation	Key features & experimental validations
a. GAN architectural frameworks			
AMP-GAN 2021	GAN (Generator & Discriminator)	Random latent vectors without explicit conditioning	Antimicrobial & cytotoxicity assays. Experimental Validation: Yes, *in-vitro* validation (Van Oort et al., 2021).
AMPGAN v2 2021	BiCGAN (Bidirectional Conditional GAN)	Binary vectors for targeting microbes & mechanisms	Metrics: Validity (95%), Novelty (94%), Uniqueness (100%). Experimental Validation: No, computational validation (Van Oort et al., 2021).
dsAMP & dsAMPGAN 2024	CNN Attention, BiLSTM, transfer learning models		AMP prediction. Metrics: Accuracy (95%), F1 (0.94). Experimental Validation: No, computational validation (Zhao et al., 2024).
FBGAN 2024	GAN, & ESM-2	Controlled & conditioned generation	Antimicrobial, haemolytic and cytotoxicity assays. Metrics: AUROC (0.92).Experimental Validation: Unclear (Chung et al., 2024; Zervou et al., 2024).
GAC-BiTCNN-AMP 2026	GAN alongside capsule networks, bidirectional temporal CNNs with ProtTrans-T5, ESM-2 embeddings & UniRep. PsePSSM-DCT & SHAP	Primarily generative augmentations alongside AMP predictions	Accuracy 97.42%, MCC 0.92 in cross-validation alongside independent accuracy 95.32% and MCC 0.91. Experimental Validation: No, computational validation (Ali et al., 2026).
WGAN-GP 2023	WGAN-GP	AI4AMP & classifiers for *in-silico*	Predicted novel peptides using methods like PC6, based on physicochemical properties. Experimental Validation: Unclear (Lin et al., 2023).
Multi-CGAN 2024	cGAN	Conditional generation	Antimicrobial & cytotoxicity assays. Experimental Validation: No, computational validation (Yu et al., 2024).
b. VAE & latent spaces-based frameworks			
CLaSS (Controlled Latent Attribute Space Sampling) 2023	WAE	Discriminator-guiding filtering	*In-vivo* models using Antimicrobial, haemolytic and cytotoxicity assays. Metrics: Precision (90%) for desired sampling. Experimental Validation: Yes, *in-vitro* & *in-vivo* validation (Wang et al., 2023).
LSSAMP 2023/2025	Vector quantized VAE	Latent space sampling	Predict Antimicrobial, haemolytic and cytotoxicity assays. Metrics: Accuracy (91.7%). Experimental Validation: Yes, *in-vitro* validation only (Wang et al., 2023; Yan et al., 2025).
Pathogen-targeted programmable AMPs 2025	Conditional VAEs, conditional diffusion models & pathogen-specific MIC predictors	Predicted physicochemical properties, targeting *E. coli* & *S. aureus*	Pathogen-specific efficacy & identified two “star” AMPs per target species with predicted antibacterial activity, toxicity & haemolysis profiles. Experimental Validation (limited): Yes, *in-vitro* validation (Zhao et al., 2026).
PepVAE 2021	VAE	Latent space sampling	Microbial activity. Metrics: Validity (>95%), Novelty (80%. Experimental Validation: Yes, *in-vitro* validation only (Dean and Walper, 2020).
c. Diffusion-based framework			
AMP-Diffusion 2025/2024	Structurally guided diffusion model	Positive learning, using discriminator-guiding filtering	Microbial & cytotoxicity assays. Sequence validity (97%). Experimental Validation: Yes, *in-vivo* validation (Torres et al., 2024; Luo et al., 2025).
Diff-AMPs 2024	Diffusion	Discriminator-guiding filtering	Metrics: AUROC (0.94). Experimental Validation: No, computational validation (Wang et al., 2024).
ProDCARL 2026 (pre-print)	RL-aligned diffusion model having EvoDiff OA-DM 38M backbone	Predicted AMP activity and low toxicity using classifier-derived rewards	Mean predicted score (0.081 → 0.178), hit rate (6.3% using pAMP >0.7 and pTox <0.3), diversity retained with 1 − mean pairwise identity = 0.929. Experimental Validation: No, computational validation (Sheng et al., 2026).
ProT-Dif 2024	Protein language diffusion	Condition generation with discriminator-guided filtering. Positive-only learning	*De novo* generation of novel AMP sequences. Metrics: Validity (98.3%), Novelty (99%).Experimental Validation: Yes, *in-vitro* validation only (Wang et al., 2024).
MMCD 2024	Diffusion model based on discriminators	Conditional generation, contrastive learning	Predict microbial, haemolytic &cytotoxicity assays. Experimental Validation: No, computational validation (Wang et al., 2024b; Lai et al., 2025).
d. Multi-objective evolutionary or genetic optimization models			
AMPEMO 2023/2025	Genetic algorithm	Discriminator-guiding filtering	Antimicrobial activity. Experimental Validation: Unclear (Liu et al., 2023; Wang et al., 2025b).
MODAN 2023	GAN & RL	Bayesian optimization	Antimicrobial, haemolytic assays. Multi-objective score improvement >30% over baseline. Experimental Validation: Unclear (Murakami et al., 2023).
MOQA Multi-CGAN QMO (Multi-Objective Quantum Annealing) 2024	Binary VAE, Multi-generator CGAN	D-Wave quantum annealer, with conditional generation	*In-vivo* models to predict Antimicrobial, haemolytic and cytotoxicity assays. Experimental Validation: Unclear (Dongrui et al., 2024).
M3-CAD 2024	cVAE	Conditional generation using discriminator-guided filtering	*In-vivo* models to predict Antimicrobial, haemolytic and cytotoxicity assays. Experimental Validation: Unclear (Wang et al., 2024a).
QMO 2022	WAE	Zero-order optimization with gradient descent	Optimized materials, i.e., drug-likeness & solubility. Experimental Validation: No, computational validation (Hoffman et al., 2022).
e. Transformers or an RNN-based hybrid framework			
AMPGen 2025	Autoregressive diffusion model, XGBoost discriminator, & STM	MSA-conditional generation	Metrics: Validity (94%), Novelty (96%). Experimental Validation: Yes, *in-vitro* validation only (Jin et al., 2025).
AMPTrans-LSTM 2023	LSTM & transformers	Learning using protein databases	Antimicrobial activity. Experimental Validation: No, computational validation (Mao et al., 2023).
CoLPAT-AMP 2026	Transformer-based AMP generators	Property-aware	Designed for novel AMPs with desired properties. Experimental Validation: No, computational validation (Salimi and Lee, 2026).
HydrAMP 2023	cVAE-GAN hybrid	Conditional generation	Antimicrobial, haemolytic assays. Metrics: AUROC (0.93). Experimental Validation: Yes, *in-vitro* validation (Szymczak et al., 2023).
f. Large language models (LLMs)			
CFlowAMP 2026	ESM-2 protein language model	Property-based generation in latent peptide spaces	Designed for property-controllable *de novo* AMP generation. Experimental Validation: No, computational validation (Chen et al., 2026).
ChatGPT prompt-oriented multifunctional AMPs 2026	Prompt-engineered LLMs	Prompt-based AMPs having single-, dual-, triple- and quadruple-functional outputs	*In-silico* accuracy >0.9 for single, dual-functional AMPs and 0.8 for triple & quadruple designs. Experimental Validation: No, computational validation (Sowers et al., 2026).
MAC-AMP 2026	Closed-loop multi-agent LLMs	Multi-objective activity, AMP-likeness, toxicity, novelty, and structure	Autonomous task-to-design frameworks, and reported AMP generators across activity, toxicity, novelty, and structural reliability. Experimental Validation: No, computational validation (Zhou et al., 2026).
PepGen-FB 2026	Feedback-driven generative frameworks alongside curriculum learning-guided multi-purpose optimization	Multi-faceted AMP generation alongside feedback optimization	Data-centric, feedback-driven frameworks for multi-purpose AMP generation for activity, safety & functional optimization. Experimental Validation: No, computational validation (Xiao et al., 2026).
g. Other generative models			
Buehler et al. 2023	GNN	Conditional generation	Physicochemical properties prediction. Experimental Validation: No, computational validation (Buehler, 2023).
Cao et al. 2020/2021	GAN	Discriminator-guiding filtering	Antimicrobial activity. Experimental Validation: Yes, *in-vitro* validation (Cao et al., 2023).
Capecchi et al. 2023	RNN	Positive learning, using discriminator-guided filtering	Antimicrobial and haemolysis assays. Experimental Validation: Yes, *in-vitro* validation (Capecchi et al., 2021).
Dean et al. 2021	VAE	Latent space sampling	Antimicrobial activity. Experimental Validation: Yes, *in-vitro* validation (Dean and Walper, 2020).
Ghorbani et al. 2022 (pre-print)	VAE	Standard VAE without variational attention	AMP prediction. Metrics: AUROC 0.90.Experimental Validation: No, computational validation (Ghorbani et al., 2022).
Jain et al. 2022	GFlowNets & active learning	Active learning	AMP prediction. Experimental Validation: No, computational validation (Jain et al., 2022).
Pandi et al. 2023	VAE	Discriminator-guiding filtering	Antimicrobial, haemolytic and cytotoxicity assays. Metrics: Validity 96%. Experimental Validation: Yes, *in-vitro* validation only (Pandi et al., 2023).
Renaud & Mansbach 2023	VAE	Latent space sampling	Physicochemical properties prediction. Experimental Validation: No, computational validation (Renaud and Mansbach, 2023).
Zeng et al. 2024 (pre-print)	PLM & BERT	Discriminator-guiding filtering	Metrics: Accuracy 92%. Experimental Validation: No, computational validation (Zeng et al., 2024).

Amongst these, GAN-based methods have good capabilities for the *de novo* generation of structurally and functionally novel and active AMPs (Dong et al., 2025b; Lai et al., 2025). Bidirectional and conditional GAN architectural frameworks have enabled iterative feedback loops between generators and discriminators, incorporating auxiliary classifiers and regressors to implement physicochemical constraints alongside antimicrobial properties (Vasconcelos et al., 2024; Zhao et al., 2024; Dong et al., 2025b; Lai et al., 2025). These approaches have produced novel AMPs having broad-spectrum activity against multidrug-resistant pathogens exhibiting lower MICs and minimal haemolytic activity (Vasconcelos et al., 2024; Zhao et al., 2024; Lai et al., 2025). However, GAN models are sensitive to training instability and usually require careful fine-tuning, necessitating incorporation alongside downstream predictive models for guiding sequential optimization (Zervou et al., 2024; Zhao et al., 2024; Lai et al., 2025).

Variational autoencoders (VAEs) have allowed complementary approaches by encoding sequences into continual latent spaces, enabling controlled interpolations for property-guided AMP design (Zhao et al., 2025). Conditional VAEs can incorporate physicochemical features, i.e., charge, molecular weight, and hydrophobicity parameters, into the design and generation processes, with targeted structural and functional profiles (Zhao et al., 2025). VAEs have provided interpretable and stable latent frameworks compared to GANs; their outputs usually require filtering and validation to ensure their biological relevance (Xie et al., 2024; Zhao et al., 2025). Diffusion-based approaches have recently evolved as stronger generative tools, iteratively processing random noises into biologically relevant AMPs (Wang et al., 2024; Wang et al., 2024).

These models, in combination with pretrained protein-based LLM embeddings, have demonstrated higher success rates for generating potent and diverse AMPs with better *in-vivo* efficacy and safety profiles (Wang et al., 2024; Wang et al., 2024). Generative performance metrics must be interpreted cautiously because computational outputs such as validity, novelty and predicted bioactivity cannot establish biological and translational generalizability, especially when reward functions and discriminative filters inherit overlapping available AMP datasets; these concerns are thoroughly discussed further in section 1.3.3.

Collectively, generative AI frameworks have enabled the transition from passive screening to active mining and optimization of sequential spaces, accelerating the novel AMPs discovery (Lai et al., 2025; Lai et al., 2025). However, the translation impacts depend on their incorporation of discriminative inputs, structural verification, and Iterative experimentation (Lai et al., 2025; Lai et al., 2025).

Most efficient strategies strongly rely on multi-model and hybrid pipelines, where generative AI models *de novo* design and generate non-natural and novel AMPs, learn distributions within peptide sequential spaces and propose previously unseen sequences, thereby shifting computational discovery towards *de novo* AMP designs (Lai et al., 2025). Generative models are increasingly being combined alongside hybrid frameworks, where AMPs are evaluated using structural, novelty, bioactivity, stability and toxicity predictors (Lai et al., 2025) (**Table 3**). Therefore, hybrid approaches can offer attractive routes towards multi-purpose AMP optimization, although they require independent validations, model calibration, followed by experimentally validated feedback (Lai et al., 2025) (**Table 3**).

**Table 3:** Comparative characteristics of discriminative, generative and hybrid AI approaches for AMP discovery and optimization.

AI paradigm	key strengths	Key limitations	AMP applications	Role within multimodel pipelines
Discriminative models	High-throughput ranking and classification of pre-existing sequences (Zhao et al., 2024; Lin et al., 2025). It requires straightforward training and benchmarking, which exploits evolutionary, physicochemical, and structural representations. These models are suitable for binary, multilabel and multiclass regression tasks, which are computationally efficient once trained (Zhao et al., 2024; Lin et al., 2025).	These models are inherently unable to explore sequential spaces beyond supplied candidates and are dependent on training-set quality and negative-set construction (Noor et al., 2025; Szymczak et al., 2025; Zhang et al., 2025). They are susceptible to sequence redundancy, class imbalance and data leakage; therefore, their performance may deteriorate on taxonomically and structurally distant AMPs (Noor et al., 2025; Szymczak et al., 2025; Zhang et al., 2025).	Binary AMP classification; stability/protease susceptibility prediction; MIC estimation; cytotoxicity and haemolysis screening (Zhao et al., 2024; Lin et al., 2025).	Decision and screening layer, which helps to filter larger datasets and assigns safety, bioactivity, potency, and developability scores before wet-lab evaluation (Zhao et al., 2024; Lin et al., 2025).
Generative models	Generate *de novo* AMPs beyond known libraries and explore larger sequence spaces and permit property-aware generation (Van Oort et al., 2021; Ghorbani et al., 2022; Lin et al., 2023; Zervou et al., 2024; Guan et al., 2025). They also enable sequential diversification and scaffold redesigns (Van Oort et al., 2021; Ghorbani et al., 2022; Lin et al., 2023; Zervou et al., 2024; Guan et al., 2025). Latest diffusion and LLMs learn complex sequence distributions without explicating handcrafted rules (Van Oort et al., 2021; Ghorbani et al., 2022; Lin et al., 2023; Zervou et al., 2024; Guan et al., 2025).	Generated sequences might not be active, safe, and novel (Van Oort et al., 2021; Ghorbani et al., 2022; Lin et al., 2023; Zervou et al., 2024; Guan et al., 2025). These models are prone to training-data bias, mode collapse or memorization; property control can be imperfect and computational metrics frequently exceed the depth of wet-lab validation (Van Oort et al., 2021; Ghorbani et al., 2022; Lin et al., 2023; Zervou et al., 2024; Guan et al., 2025).	*De novo* generation and diversification of known AMP scaffolds. Conditional AMP generation based on charge, length and bioactivity (pathogen-directed designs), generation of broad-spectrum candidates by exploring previously untested sequence spaces (Van Oort et al., 2021; Ghorbani et al., 2022; Lin et al., 2023; Zervou et al., 2024; Guan et al., 2025).	The exploration layer will help to propose new AMPs that are passed through filters for novelty, structure, bioactivity, cytotoxicity, and stability (Van Oort et al., 2021; Ghorbani et al., 2022; Lin et al., 2023; Zervou et al., 2024; Guan et al., 2025).
Hybrid AI approaches	These approaches combine the exploratory capacity of generative AI with the selectivity of discriminative prediction, enabling simultaneous optimisation (e.g., novelty, stability, potency, toxicity) and reducing the number of generated AMPs that require experimental testing, thereby best aligning with end-to-end precise AMP engineering (Noor et al., 2025; Sun, 2025).	These are computationally and architecturally complex, and prediction errors can propagate between modules (Noor et al., 2025; Sun, 2025). Performance metrics depend on compatibility and calibration of individual models. Moreover, closed-loop optimization can exploit model biases, and prospective wet-lab validation remains limited (Noor et al., 2025; Sun, 2025).	Suitable for multi-purpose AMP designing. Activity–toxicity trade-off optimization by adapting pathogen-specific AMP engineering (Noor et al., 2025; Sun, 2025). Optimization of stability and selectivity by iteratively design–predict–refine workflows (Noor et al., 2025; Sun, 2025).	Introduction of an integration and optimization layer will link sequential generation, discriminative screening, and structural evaluations into iterative decision frameworks before biological validation (Noor et al., 2025; Sun, 2025).

On the contrary, discriminative models assess whether predicted peptides possess antimicrobial properties from pre-existing resources and learn quantitative relationships between predefined biological endpoints and peptide representations, therefore suitable for classification, regression and AMP prioritization (Lai et al., 2025) (**Table 3**). Convergence of both generative and predictive paradigms demonstrates critical steps toward precision-driven AMPs design and generation to address the accelerating global AMR burden (Li et al., 2025; Mittal et al., 2025; Wang et al., 2025; Zervou et al., 2025).

#### 1.2.3 Dataset overlapping, negative-set construction and over-generalization

One of the key limitations associated with discriminative models; independent predictors are developed and evaluated using overlapping databases (Agüero-Chapin et al., 2023). Mostly, positive AMPs are repeatedly extracted from commercially available repositories, followed by diverse combinations of sequence-length filtering, redundancy reduction and train–test partitioning (Agüero-Chapin et al., 2023). Consequently, comparisons amongst models cannot be interpreted as evaluations based on independent biological information (Agüero-Chapin et al., 2023).

Importantly, random splitting places identical and evolutionarily related AMPs across test and training partitions, exploiting sequential similarities rather than learning determinants of bioactivity (Zhao et al., 2026). They can inflate performance metrics, explaining why models report excellent internal benchmark performances but can generalize less effectively to AMPs from previously unexplored families and ecological environments (Zhao et al., 2026). Thus, complex architectures must not be interpreted as biologically superior when sequential overlapping, benchmark provenance, and homology between evaluation and training datasets have not been controlled (Wang et al., 2025).

Negative class has represented one of the equally important sources of bias (Yang and Xu, 2024). Experimentally validated inactive AMPs are scarce because unsuccessful AMPs have not been reported systematically (Yang and Xu, 2024). Fewer AMP classifiers have been constructed using negative datasets from computationally convenient and reliable sources (Yang and Xu, 2024). Absence of AMP annotations does not support experimental evidence of microbial inactivity. If artificially negative outputs differ from positive AMPs depending on amino-acid composition, length, charge, and cellular origin, AMP classifiers can learn these dataset-construction artefacts rather than biologically active determinants (Zhao et al., 2026). These concerns have been extended to specialized safety classifiers, where incomplete non-haemolytic sequences and heterogeneous assay conditions can influence labels, thereby apparently affecting model performance (Yang and Xu, 2024).

Performance metrics across AMP prediction models must not be interpreted as directly comparable measures for superiority (Agüero-Chapin et al., 2023). Although several studies have reported F1-score, accuracy, AUROC, and MCC values exceeding 90%, these outcomes are derived from heterogeneous datasets, sequence-redundancy thresholds, negative-set definitions, and validation strategies (Zhao et al., 2026). Moreover, these performance metrics capture different aspects of predictive behaviour; therefore, they cannot be ranked interchangeably across studies. Accordingly, the performance metrics summarized in **Table 1**–**9** must be interpreted as within-study evidence of model performance only. Direct superiority claims can be justified when competing models have been evaluated on the same independent datasets using redundancy control, identical preprocessing, decision thresholds and evaluation metrics (Zhao et al., 2026).

**Table 4:** *In-silico* haemolytic predicted models that assess the safety of novel AMPs.

Models & year	Architectural framework	Performance metrics	Key findings & experimental-based evidence
AMPDeep 2022	Transfer learning & DL with protein LLM.	Accuracy =90%, AUC =0.94	Crucial step toward representation learning in AMP safety & used pretrained embeddings. Experimental Validation: No, computational validation (Salem et al., 2022).
CNN-based AMP haemolysis predictor 2024	DL with CNNs.	Accuracy =88–92%	Reinforced the end-to-end sequence models to improve haemolysis prediction. Experimental Validation: No, computational validation (Abdelbaky et al., 2024).
ConsAMPHemo 2025	Classical & hybrid workflows with binary classification alongside concentration-regression frameworks.	AUC =0.95	Both classification and haemolysis concentration prediction. Experimental Validation: No, computational validation (Xie et al., 2025).
Enhanced DL haemolysis predictor 2024	DL sequence-analysis frameworks using learned peptide representations.	Accuracy 94.60%, Precision 94.10%, Recall 95.20%, F1-score 94.65%, ROC-AUC 0.973	No new peptide synthesis reported. Experimental Validation: No, computational validation (Abdelbaky et al., 2024).
HemoPred 2017	Classical ML webserver.	Accuracy=95%, MCC reported	Early benchmark haemolysis classifier. Experimental Validation: No, computational validation (Win et al., 2017).
HLPpred-Fuse 2020	Multi-feature fusion ML.	AUC =0.94	Improved robustness over earlier single-model tools. Experimental Validation: No, computational validation (Hasan et al., 2020).
HAPPENN 2020	DL with neural-network classifiers.	Accuracy =85–90%	Strengthened sequence-only deep classification. Experimental Validation: No, computational validation (Timmons and Hewage, 2020).
Haemolytic potency prediction for chemically modified peptides 2020	Classical ML, built from 2D/3D descriptors and fingerprints.	Quantitative HC50 predictions.	Prediction toward potency-aware haemolysis modelling in modified peptide spaces. Experimental Validation: No, computational validation (Kumar et al., 2020).
HemoNet 2021	DL with CNNs & RNNs.	AUC =0.93	Reported a stronger AUC-ROC than HemoPI/HemoPred. Experimental Validation: No, computational validation (Kumar et al., 2020).
HemPepPred 2025	Ensemble ML & PLM-derived features, using ML with protein-LLM.	State-of-the-art performances.	Next-generation trend of combining PLM embeddings. Experimental Validation: No, computational validation (Li et al., 2025).
HemoDL 2024	Ensemble DL with a double-ensemble learning strategy.	AUC =0.95	Engineered ensemble designs even in the deep-learning era. Experimental Validation: No, computational validation (Yang and Xu, 2024).
HemoFuse 2024	Multi-feature fusion networks with multi-head cross-attention.	Accuracy 95.33%, Sensitivity 94.00%, Specificity 96.67%, MCC 0.907, AUC 0.982	No direct wet-lab validation reported. Experimental Validation: No, computational validation (Zhao et al., 2024).
LLM toxicity prediction for AMPs 2024	LLMs, transformers & GPT prompting	Emerging benchmark study.	General-purpose LLMs for peptide toxicity prediction. Experimental Validation: No, computational validation (Orsi and Reymond, 2024).
ML-guided non-haemolytic peptide discovery 2020	ML & design workflow.	Experimental validation	Linked prediction to prospective peptide design. Experimental Validation: No, computational validation (Plisson et al., 2020).
Quantitative regression model 2024	Regression ML, predicting hemolytic concentrations.	RMSE/R² reported	Moved beyond binary labels toward quantitative haemolysis modelling. Experimental Validation: No, computational validation (Karasev et al., 2024).
Prediction of hemolytic peptides and HC50 2025	Hybrid ML, regression plus classification for HC50 prediction.	Classification alongside HC50	HC50-level quantitative prediction. Experimental Validation: No, computational validation (Rathore et al., 2025).
tAMPer 2024	Transformer-based safety model	High AUC reported.	Structure-aware peptide toxicity prediction to improve toxicity estimation. Experimental Validation: No, computational validation (Ebrahimikondori et al., 2024).
ToxGIN 2024	Graphs DL.	AUC =0.94	General peptide toxicity model. Experimental Validation: No, computational validation (Yu et al., 2024).

**Table 5:** *In-silico* models that assess the cytotoxicity profiles of predicted novel AMPs.

Models & year	Architectural framework	Performance metrics	Key findings & Prospective experimental validation
ATSE 2021	DL (GNN, attention), structural, evolutionary & sequence integrations.	Accuracy=93.7%, MCC=0.87 AUC=0.96	Enhanced prediction using graph-based representations. Experimental Validation: Computational benchmarking only (Wei et al., 2021).
ClanTox 2019	ML Sequence-based classifiers.	AUC =0.90 Accuracy=85–90%	Applied to animal toxin classification. Experimental Validation: Computational benchmarking only (Cole and Brewer, 2019).
DeepTox 2016–ongoing	DL, multilayer neural networks.	ROC-AUC=0.84	Widely validated for chemical toxicity. Experimental Validation: Computational benchmarking only (Mayr et al., 2016).
LLM toxicity prediction for AMPs 2024	GPT & LLM prompting.	Competitive with classical ML	GPT-3 performed not reproducibly for activity prediction and haemolysis, taken as a proxy for toxicity. Experimental Validation: Computational benchmarking only (Orsi and Reymond, 2024).
NNTox 2019	ML (Neural Network) with Gene Ontology and functional annotations.	Accuracy=84.5% AUC=0.91	Improved functional toxicity prediction. Experimental Validation: Computational benchmarking only (Jain and Kihara, 2019).
ProTox-II 2018–ongoing	ML, Descriptor-based toxicity predictions.	Accuracy=76% ROC-AUC=0.84	Extensively validated for drug candidates. Experimental Validation: Computational benchmarking only (Banerjee et al., 2018).
Structure-aware peptide toxicity model/tAMPer 2024	DL & structure-aware embeddings	AUC=0.94	Experimental Validation: *In-vitro* validation using pig RBCs (Ebrahimikondori et al., 2024).
ToxinPred 2013	ML (SVM) AAC, dipeptide composition, motif-based features.	Accuracy =89.5%, MCC =0.80 AUC=0.94	Widely validated on peptide datasets; foundational toxicity predictor. Experimental Validation: Computational benchmarking only (Gupta et al., 2013).
ToxClassifier 2016	ML, feature-based classifiers for venom toxins	Accuracy =95%	Differentiates venom vs non-toxic proteins. Experimental Validation: Computational benchmarking only (Gacesa et al., 2016).
TOXIFY 2019	DL, sequence-based neural network	Accuracy=95.0% F1-score=0.93 AUC=0.97	Validated on venom protein datasets. Experimental Validation: Computational benchmarking only (Cole and Brewer, 2019).
ToxDL 2020	DL, sequence & domain embeddings (DL architecture)	ROC-AUC=0.83–0.88	Demonstrated improved classification of protein toxicity. Experimental Validation: Computational benchmarking only (Villegas-Morcillo et al., 2020).
ToxinPred2 2022	Hybrid ML, BLAST, motifs & ML ensemble (RF, SVM, XGBoost)	Accuracy=96.5% MCC=0.93 AUC=0.98	AUC=0.99; validated on large SwissProt datasets. Experimental Validation: Computational benchmarking only (Sharma et al., 2022).

**Table 6:** *In-silico* MIC regression models to predict efficacy against drug-resistant pathogens.

Models & year	Architectural framework	Performance metrics	Key findings & experimental evidence
ANIA 2026	Inception & attention MIC predictions.	High MIC prediction accuracy.	Not reported. Experimental Validation: Computational benchmarking only (Chiu et al., 2026).
Amino acid–based activity prediction 2014	Classical ML with residual contribution scorings & sequence optimization.	MIC hit enrichment reported.	Experimental Validation: *In-vitro* & *in-vivo* validations (Wu et al., 2014).
AMP regression benchmarking 2021	ML + DL comparison CNN, RF, XGBoost, Elastic Net for MIC predictions.	R²=0.45–0.72 RMSE improved over baselines.	Demonstrated GB/XGBoost superiority for MIC regression. Experimental Validation: *In-vitro* validation (Dean et al., 2021).
AMPredictor & GAN pipeline 2025	Generative; graph DL GAN generator & GCN MIC regressors.	RMSE 0.5348 Higher hit rates.	Experimental validation: 24 peptides validated via *in-vivo* efficacy (Dong et al., 2025b).
BERT-AmPEP60 2025	Transformers with fine-tuned ProtBERT & regression heads.	Accuracy=95%, AUC=0.97 MSE=0.266 (*E. coli*) Pearson=0.796	Strong generalization. Experimental Validation: Computational benchmarking only (Cai et al., 2025).
BroadAMP-GPT 2025	Foundation & GPT model	Broad-spectrum activity.	Predicted antimicrobial activity. Experimental validation: *In-vitro*/potentially *in-vivo*, requires paper-specific qualification (Li et al., 2025).
CNN-based AMP activity prediction 2024	DL with CNN-based sequence & structural features.	Accuracy=92–95%, AUC=0.95	Improved AMP activity prediction vs ML baselines. Experimental Validation: Unclear (Mitra and Singh, 2023).
DeepPIE/ProtBERT-based MIC frameworks 2024–2025	Transformer DL with PLM embeddings & neural regression layers.	R²=0.70–0.82 MAE improved vs classical QSAR	Improved MIC prediction using contextual embeddings. Experimental Validation: Unclear (Wang et al., 2025).
ESKAPEE-MICpred 2024	Transfer learning & ensemble pathogen-aware predictions integrating sequence & genomic features	RMSE low Pearson r=>0.80	Designed for WHO-priority pathogens; improved generalization. Experimental Validation: Computational benchmarking only (Sharma et al., 2024).
Ensemble MIC predictor 2024	Deep ensemble with BiLSTM, CNN, multi-branch & genomic features.	AUC=0.96 R²=0.79 Pearson 0.756–0.802	Predicted antimicrobial activity. Experimental Validation: Computational benchmarking only (Chung et al., 2024).
LLM foundation AMP model 2025	LLM peptide generators.	Potent candidates against resistant pathogens	*De novo* designing, generation and antimicrobial activity prediction. Experimental Validation: *In-vitro* & *in-vivo* validations (Wang et al., 2025d).
MBC-Attention 2023	DL with multi-branch CNN & attention mechanisms.	R²=0.84 PCC=0.92 Pearson 0.775 RMSE 0.533	*E. coli* MIC prediction. Experimental Validation: Computational benchmarking only (Yan et al., 2023).
PepVAE 2021	Generative DL, ML regression, VAE & downstream regressors (GB, RF).	R^2^ = 0.73 RMSE=0.50	Experimentally validate AMPs. Experimental Validation: *In-vitro* validation (Dean et al., 2021).
QSAR-based AMP activity models 2006–2010	Classical ML with physicochemical descriptors & regression (QSAR).	R² 0.55–0.75	Early quantitative activity predictions with limited datasets. Experimental Validation: Unclear (Rajakumar et al., 2006).

**Table 7:** *In-silico* novelty filters that assess novelty at the genomic and protein-sequence levels.

Models & year	Architectural framework	Performance metrics	Key findings & experimental validation
BLAST/BLASTP/ tBLASTn 2006-2009	Heuristic local alignment with seed-and-extend architecture. Protein-to-translated-nucleotide local alignment across six frames.	High sensitivity with standard benchmark references & faster than Smith-Waterman with strong recall.	Foundational sequence-similarity baseline with sensitive protein novelty checks. Improved translated-search reliability via composition-based statistics & suited to genomic novelty mapping. Experimental Validation: Computational benchmarking only (Gertz et al., 2006; Camacho et al., 2009).
CD-HIT 2012	Greedy incremental clustering with word-count filtering.	More than 100 times faster than all clustering.	Fast redundancy reduction & useful to collapse near-identical candidates before novelty analysis. Experimental Validation: Computational benchmarking only (Fu et al., 2012).
DIAMOND v2 2021	Double indexing, spaced seeds, vectorized extension, HPC-distributed protein alignments.	Up to 20,000× faster than BLASTX.	BLAST-like sensitivity with 80-fold BLASTP speedup in ultra-sensitive modes & 18 h nr-vs-UniRef50 on 520 nodes. Experimental Validation: Computational benchmarking only (Buchfink et al., 2021).
Foldseek 2024	Fast structural search using structure-to-sequence style encoding.	>10,000× faster than structural aligners, high sensitivity comparable to TM-align/DALI class tools.	Enabled structural novelty screening when sequence similarity was weak, but fold similarity remained high. Experimental Validation: Computational benchmarking only (van Kempen et al., 2024).
HMMER 2011	Profile-HMM–based remote homology search.	Higher remote-homology sensitivity than pairwise alignment with trusted E-value frameworks.	Widely used for remote family assignment beyond pairwise sequence similarity. Experimental Validation: Computational benchmarking only (Finn et al., 2011).
InterProScan 5 2014	Integrated protein-signature and domain-scanning frameworks.	Broad functional annotation coverage with millions of sequences.	Genome-scale functional & domain classification helped reject candidates from known families. Experimental Validation: Computational benchmarking only (Jones et al., 2014).
MMseqs2 2017	Sensitive, indexed protein search optimized for massive datasets.	100–400× faster than BLAST, high sensitivity close to PSI-BLAST.	Reported sensitivity better than PSI-BLAST at >400× its speed. Experimental Validation: Computational benchmarking only (Steinegger and Söding, 2017).

**Table 8:** *In-silico* models that evaluate stability against non-enzymatic and enzymatic degradation.

Models & year	Architectural framework	Performance metrics	Key findings & experimental validation
DeepCleave 2020	Deep learning protease cleavage prediction.	AUC=0.93	Outperformed in predicting caspase and matrix metalloprotease substrate-cleavage sites. Experimental Validation: Computational benchmarking only (Li et al., 2020a).
MPCutter 2025/26	Multi-protease cleavage prediction model.	Outperformed existing general tools on independent tests.	High-throughput prediction of protease-specific substrates and exploration of protease proteolytic events. Experimental Validation: Computational benchmarking only (Wang et al., 2025).
PROSPER 2012	ML has an integrated feature-based cleavage-site prediction using sequence-derived features across multiple proteases.	AUC=0.84–0.96 Accuracy =80–90%	Reported comprehensive multi-protease cleavage prediction & established ML-based substrate prediction as a practical workflow. Experimental Validation: Computational benchmarking only (Song et al., 2012).
PROSPERous 2018	ML has a logistic-regression-based high-throughput prediction using multiple scoring functions and pairwise feature combinations for 90 proteases.	AUC=0.87–0.98 MCC improved over PROSPER.	Improved accuracy and broader protease coverage than earlier tools & state-of-the-art high-throughput cleavage prediction at publication. Experimental Validation: Computational benchmarking only (Song et al., 2018).
ProsperousPlus 2023	Ensemble ML & AutoML cleavage prediction platform.	State-of-the-art prediction across 110 protease types	Predicted substrate cleavage sites & facilitated the use of the rapidly accumulating substrate cleavage data to train, empirically assess and deploy predictive models. Experimental Validation: Computational benchmarking only (Li et al., 2023).
Procleave 2020	ML with protease-specific prediction combining sequence & structural information with LOWESS-smoothed structural determinants.	AUC=0.90 Accuracy=85–92%	Demonstrated improved cleavage-site prediction by incorporating 3D structure, which is directly relevant to peptide stability design. Experimental Validation: Computational benchmarking only (Li et al., 2020b).
PlifePred 2018	ML has an SVM-based peptide blood half-life prediction using chemical descriptors from experimentally measured natural and modified peptides.	MCC=0.80, Accuracy =85% AUC =0.91	Built from peptides with experimentally determined half-lives in mammalian blood & useful for pre-screening pharmacological persistence. Experimental Validation: Computational benchmarking only (Mathur et al., 2018).
panCleave 2023	ML with a pan-protease random forest model for computational proteolysis and encrypted-peptide discovery.	AUC=0.90	Used to mine encrypted antimicrobial fragments with downstream peptides that showed antimicrobial activity with experimental and mouse-model validation in the discovery workflow. Experimental Validation: *In-vitro* & *in-vivo* validations (Maasch et al., 2023).
PeptideCutter 2025	Rule-based cleavage-site mapping for proteases and chemicals.	Deterministic cleavage mapping.	Long-standing practical screen for identifying cleavage hotspots in peptide sequences. Experimental Validation: Computational benchmarking only (Turalić et al., 2025).
UniZyme 2025	Transformer & PLM-based unified protein cleavage predictor with enzyme active sites	Strong generalization	High accuracy for predicting cleavage sites across a range of proteolytic enzymes. Experimental Validation: Computational benchmarking only (Li et al., 2025).

**Table 9:** Molecular docking to assess and evaluate mechanisms by providing sequence-based outcomes before wet-lab validation of AMPs.

Model & year	Architectural framework	Performance metrics	Experimental validation
Classical physics-oriented molecular or ligand docking			
AutoDock 4/Vina 2004–2010	It is physics & grid-based docking, having empirical scoring & local optimization.	Vina is 100× faster than AD4 & improved pose prediction accuracy.	Widely validated & improved speed and accuracy for binding pose prediction. Experimental Validation: Computational benchmarking only (Morris et al., 2009).
AutoDock-GPU 2022	GPU-accelerated AutoDock.	Massive runtime reduction vs CPU AD4.	Facilitated large-scale virtual screening. Experimental Validation: Computational benchmarking only (Santos-Martins et al., 2021).
DOCK6 2009-2024	Anchor-and-grow incremental docking alongside grid-based scorings.	Robust enrichments for virtual screening benchmarks.	Validated for ligand discovery campaigns. Experimental Validation: Computational benchmarking only (Lang et al., 2009; Allen et al., 2015; Balius et al., 2024).
FlexAID 2015	Partial (heuristic AI). Flexible docking with surface complementarity scoring.	Competitive pose predictions & robust for induced-fit cases.	Improved performance for flexible binding sites. Experimental Validation: Computational benchmarking only (Alhossary et al., 2015).
Glide (Schrödinger) 2004–present	It is physics-based, having hierarchical docking & empirical scoring functions.	Top-tier enrichment with 65–70% success in comparative studies.	High accuracy docking benchmark performance vs other tools. Experimental Validation: Computational benchmarking only (Friesner et al., 2004).
GOLD 2003-2006	Genetic algorithm–based flexible docking.	High enrichment factors alongside reproducible docking accuracy.	Pharmaceutical structure-based drug discovery. Experimental Validation: Computational benchmarking only (Verdonk et al., 2003; Joy et al., 2006).
Surflex-Dock 2012	Incremental fragment placements alongside empirical Hammerhead scorings.	Pose predictions and enrichments during redocking studies.	Benchmarked against Glide and GOLD in datasets. Experimental Validation: Computational benchmarking only (Spitzer and Jain, 2012).
Smina 2013	Enhanced AutoDock Vina forks with customizable minimization & scoring.	Improved scoring and benchmarking utility over Vina.	ML-assisted docking workflows, including GNINA. Experimental Validation: Computational benchmarking only (Koes et al., 2013).
Physics-based structural optimization			
FlexPepDock 2017	Rosetta Monte Carlo minimization alongside peptide backbone flexibility.	Achieved near-native peptide conformations (<2 Å RMSD).	Validated experimentally based peptide–protein complexes. Experimental Validation: Computational benchmarking only (Alam et al., 2017).
RosettaLigand 2006–2015	It is physics-based. Monte Carlo sampling, energy minimisation & flexible docking.	Strong local refinement with accurate near-native poses in benchmark sets.	Accurate binding pose prediction; used in peptide–protein docking studies. Experimental Validation: Computational benchmarking only (Davis and Baker, 2009; Lemmon and Meiler, 2012; DeLuca et al., 2015).
Structure-based foundational docking			
AlphaFold2 2021	It is deep learning (transformer-based protein structure prediction).	Median backbone accuracy at the atomic level.	Near-experimental accuracy for protein structures is widely adopted in docking pipelines. Experimental Validation: Computational benchmarking only (Jumper et al., 2021).
AlphaFold3 2024	Multimodal deep learning for protein–ligands & co-complex prediction.	Great improvement for protein–ligands & complex prediction over AF2.	Outperforms classical docking in some binding predictions. Experimental Validation: Computational benchmarking only (Abramson et al., 2024).
RoseTTAFold All-Atom 2011-2021	Unified all-atom DL frameworks for proteins, ligands, nucleic acids, & complexes	All-atom biomolecular complex predictions.	Validated across protein–ligand and macromolecular benchmark datasets. Experimental Validation: Computational benchmarking only (London et al., 2011; Raveh et al., 2011; Alam et al., 2017; Badaczewska-Dawid et al., 2021).
Diffusion/Generative molecular docking			
ArtiDock 2026	It is an ML-based docking method, having deep learning scoring & structural refinement.	High enrichment and accurate pose ranking for screening-scale throughput.	Improved RMSD and binding accuracy vs classical docking. Experimental Validation: Computational benchmarking only (Voitsitskyi et al., 2026).
DiffDock 2022-26	Generative DL & diffusion model for ligand pose generation.	RMSD <2 Å success =38–45% depending on benchmark	38% success rate vs 23% traditional docking on benchmarks. Experimental Validation: Computational benchmarking only (Corso et al., 2022).
LigDockTailor 2025	It is an ML meta-model with the selection of optimal docking tools.	Improved selection accuracy & meta-docking performance gains.	Improves docking success by matching ligand properties to tools. Experimental Validation: Computational benchmarking only (Zhang et al., 2025).
AI-assisted molecular docking			
EquiBind 2022-2023	SE (Sadeeq et al., 2025)-equivariant geometric DL for direct pose predictions.	Faster orders-of-magnitude & inference than classical docking.	Benchmarked on PDBBind datasets. Experimental Validation: Computational benchmarking only (Zhang et al., 2022; Li et al., 2023).
GNINA 2021-25	CNN rescoring & docking.	Outperformed Vina on pose ranking.	Better performance in both virtual screening of active ligands and re-docking steps of co-crystallized ligands. Experimental Validation: Computational benchmarking only (Buccheri and Rescifina, 2025).
NeuralPLexer 2024	End-to-end neural complex predictions integrating ligand and protein structural representations.	Competitive complex predictions and docking accuracies.	Demonstrated performance across protein–ligand and biomolecular complexes. Experimental Validation: Computational benchmarking only (Qiao et al., 2024).
TankBind 2022	Geometric deep learning docking.	Competitive AI docking accuracy.	Integrated trigonometric constraints and protein binding-site block attention to outperform existing physics-based and DL methods in protein–ligand pose and affinity prediction. Experimental Validation: Computational benchmarking only (Lu et al., 2022).
Uni-Mol Docking 2023-2026	Transformer-based molecular representations integrated 3D geometry.	State-of-the-art binding pose & docking predictions.	Validated across molecular and docking tasks. Experimental Validation: Computational benchmarking only (Alcaide et al., 2026).
Protein-peptide molecular docking			
CABS-dock 2015	Coarse-grained flexible peptides docking using CABS protein modelling alongside Replica Exchange Monte Carlo.	Median ligand RMSD often <5 Å without prior binding-site knowledge.	Validated on larger datasets for flexible peptide docking. Experimental Validation: Computational benchmarking only (Kurcinski et al., 2015).
GalaxyPepDock 2015	Template-based docking using interaction energy & similarity optimization.	Outperformed several docking methods in benchmark datasets.	Reproduced experimental peptide–protein complexes. Experimental Validation: Computational benchmarking only (Lee et al., 2015).
HADDOCK 2010-2024	Information-driven docking integrated biophysical & biochemical restraints alongside flexible refinements in explicit solvents.	Ranked amongst the top CAPRI predictors having strong peptide–protein docking performances.	Protein–protein and peptide–protein complex validations, also including antibody–antigen systems. Experimental Validation: Computational benchmarking only (De Vries et al., 2010; Honorato et al., 2024).
HPEPDOCK 2018	Hierarchical FFT-oriented blind peptide molecular docking alongside flexible peptide refinements.	Up to 33.3% success rates, using unbound benchmark complexes.	Benchmarked against pepATTRACT and other docking tools. Experimental Validation: Computational benchmarking only (Zhou et al., 2018).
pepATTRACT 2017-2020	Coarse-grained rigid-body docking.	Competitive blind peptide docking performances & used benchmark baselines.	Validated across protein–peptide benchmark datasets. Experimental Validation: Computational benchmarking only (Schindler et al., 2015; de Vries et al., 2017; Weng et al., 2020).
PIPER-FlexPepDock 2017-2026	Global rigid-body docking followed by Rosetta FlexPepDock refinements.	High-resolution refinements alongside sub-angstrom peptide interface accuracy.	Strong CAPRI-style benchmark performances. Experimental Validation: Computational benchmarking only (Alam et al., 2017; Tsaban et al., 2022; Martins et al., 2026).

Many discriminative models are constructed using benchmark families assembled from commercially available repositories and usually supplemented with UniProt/Swiss-Prot-derived negative sequential spaces (Zhao et al., 2026). Consequently, test and training datasets contain near-identical and evolutionarily related AMPs (Zhao et al., 2026). Moreover, highly cross-validated performance metrics partly reflect recognition of AMPs, which are represented during training rather than generalization to novel AMPs (Renaud and Mansbach, 2023). Independent test datasets can provide stronger and novel outputs only when their sequences are not included in model development and are separate from training data; labelling them as “independent datasets” cannot support biological independence (Renaud and Mansbach, 2023). Predicted models must be supported by cross-validation, and benchmark families must be interpreted cautiously and evaluated on prospective metagenomics, rigorously held-out external datasets and experimentally validated candidates (Chen et al., 2025).

Future benchmarking must prioritize wet-lab validated both negative and positive AMPs, explicitly mention assay conditions and database provenance, limit cross-partition sequential redundancy using cluster-oriented splitting, and assess models on temporally separated and external datasets (Chen et al., 2025). Performance metrics must be reported against family-held-out and experimental tests, alongside traditional random splits; this would provide stronger measures of whether AI-predicted models can generalize to the novel AMP sequential spaces that are intended to discover (Chen et al., 2025).

#### 1.2.4 *In-silico* safety screening for therapeutic-index modelling

The key barriers to the clinical translation of AMPs are host-cell toxicity, reflecting their membrane-disruptive properties (Rathore et al., 2025). Consequently, cytotoxicity-predicting models have emerged as equal objectives alongside better efficacy profiles within modern computational pipeline strategies (Rathore et al., 2025). Haemolysis and cytotoxicity are major factors in later-stage AMP therapeutic failures, highlighting the need to incorporate safety-predicted models at the initial stages of AMP design rather than as downstream filters (Rathore et al., 2025). This transition has accelerated the development of AI-driven cytotoxicity models by mapping peptide sequence spaces and structural features that link them to toxicity endpoints, enabling rapid *in-silico* triaging of novel AMPs before synthesis and wet-lab validation.

##### 1.2.4.1 *In-silico* haemolytic filters

Classical ML methods used physicochemical descriptors; however, recent approaches have increasingly leveraged DL and protein-based LLM models for capturing higher-order sequential structural–cytotoxicity relationships (Abdelbaky et al., 2024) (**Figure 7**; **Table 4**). For instance, CNNs and RNNs demonstrated improved performance for predicting haemolytic activity directly by learning local motifs and from sequential representations associated with membrane disruption and toxicity properties (Abdelbaky et al., 2024). The latest architectural frameworks, i.e. tAMPer, integrated sequential embeddings from protein-LLMs (ESM-2) alongside structural features, improving peptide toxicity predictions by incorporating 3D contexts alongside primary sequential information (Ebrahimikondori et al., 2024). These advancements reflect broader transitions toward representation learning and reduce reliance on handcrafted features, improving generalisation across diverse datasets.

**Figure 7:**
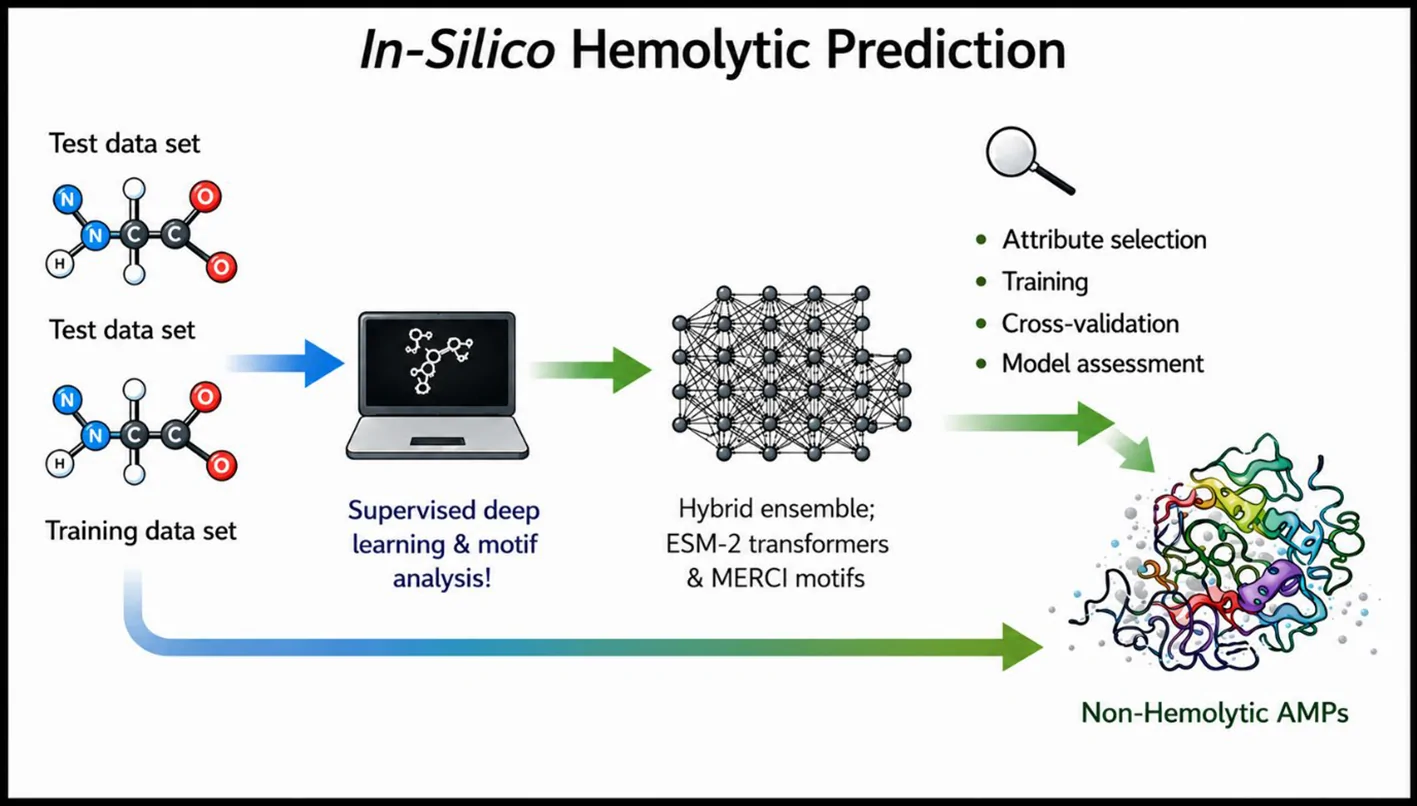
In-silico ML and DL-based haemolytic model with integrated diverse architectural frameworks, i.e., a hybrid ensemble having ESM-2, transformers, & MERCI motifs, is used to predict safety potentials. The models can assign probability scores to reflect AMP likelihoods, enabling systematic ranking of high-confidence AMPs for downstream analysis. The image was created with BioRender (https://www.biorender.com).

Haemolytic predictions have evolved from binary outcomes toward quantitative modelling. ConsAMPHemo and the latest regression-based models predict haemolytic concentrations (HC_50_), the peptide concentrations causing 50% lysis of red blood cells, and higher HC_50_ demonstrates greater blood-cell compatibility due to lower haemolytic toxicity, providing both mechanistic insights and predictive capabilities (Xie et al., 2025). Therefore, integrated pipelines have combined efficacy and safety profiling alongside binary predicted outcomes, highlighting the importance of multi-purpose learning for optimizing therapeutic indices rather than single outputs (Bhatnagar et al., 2024; Xie et al., 2025). However, certain challenges remain within dataset bias, a lack of negative data demonstrating those AMPs having haemolytic effects compared with successful, safer ones, cross-species generalization, and assay variability, underscoring the need for wet-lab validated datasets and standardized benchmarks (Bhatnagar et al., 2024; Xie et al., 2025). Discriminative AMP classifiers reporting performance of haemolysis-predicted models must be interpreted in the context of overlapping datasets and heterogeneous experimental outputs (Bhatnagar et al., 2024; Xie et al., 2025).

AMP haemolytic models have progressed from descriptor-driven binary classifiers, i.e. HemoPred with neural-network and ensemble systems (HemoNet, HLPpred-Fuse and HAPPENN) (Win et al., 2017; Hasan et al., 2020; Timmons and Hewage, 2020), to advanced graph and transfer-based learning, and multi-purpose structural-aware predictors (AMPDeep, tAMPer and ToxGIN) (Salem et al., 2022; Ebrahimikondori et al., 2024; Yu et al., 2024). These latest models not only identify haemolytic AMPs, but also estimate quantitative toxicity outputs (haemolytic concentrations), more relevant for prioritizing AMPs based on therapeutic index (the ratio of antibacterial activity to toxicity) than mere binary labels (Win et al., 2017; Hasan et al., 2020; Timmons and Hewage, 2020). These evolutionary approaches strongly support the incorporation of cytotoxicity-predicted models as front-end design constraints within AMP mining and discovery pipelines. However, reported performance metrics can be inflated by datasets overlapping with limited external validation.

##### 1.2.4.2 *In-silico* cytotoxicity filters for AMP therapeutics

Traditional wet-lab approaches for toxicity assessment are labour-intensive, costly and may rely on *in-vivo* models, reducing their scalability during early-phase discovery. Consequently, there have been sustained shifts over the past years towards computational-based toxicity predictions, leveraging (Gupta et al., 2013). Recently, DL has enabled rapid *in-silico* screening of novel AMPs (Gupta et al., 2013). Early efforts are primarily focused on similarity-driven approaches, but are limited by poor generalization (Sharma et al., 2022) (**Figure 8**; **Table 5**). ToxinPred2 has illustrated that there is a transition toward hybrid frameworks incorporating motif discovery, sequence similarity (BLAST), and ML scoring, achieving better predictive outcomes (AUC = 0.99) across various protein datasets (Sharma et al., 2022).

**Figure 8:**
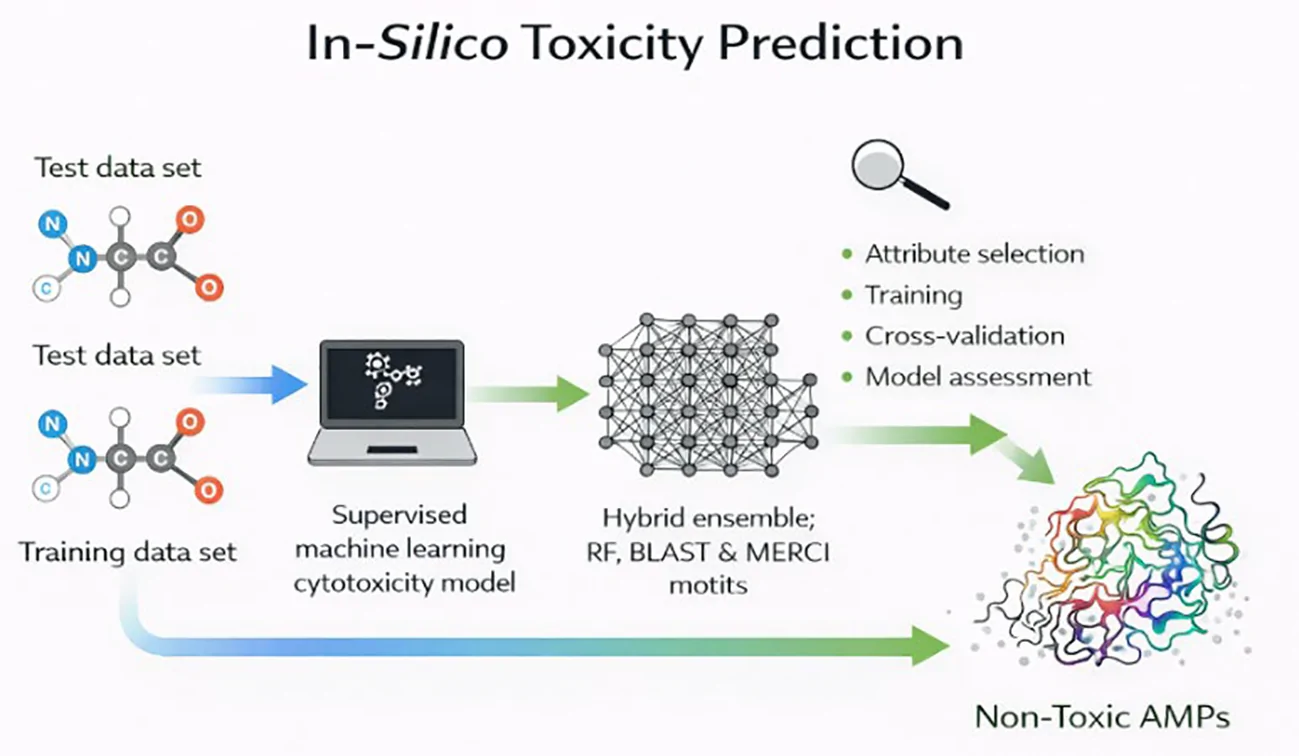
In-silico ML and DL cytotoxicity prediction models integrate diverse architectural frameworks, i.e., hybrid ensembles having RF, BLAST & MERCI motifs, to predict safety potentials. The models usually assign probability scores to reflect AMP likelihoods, enabling systematic ranking of novel AMPs for downstream analysis. The image was created with BioRender (https://www.biorender.com).

Toxicity-predicting models have evolved from feature-engineered classical ML (SVM using motif features and amino acid composition) to representation-oriented learning frameworks (Jain and Kihara, 2019). ToxinPred demonstrated sequence-derived features that effectively distinguished toxic from non-toxic AMPs, but was limited to constrained datasets and shorter peptides (Gupta et al., 2013). Advanced models (ToxDL and TOXIFY) integrated evolutionary information, gene ontology, and deep neural architectural frameworks to enhance generalization across diverse protein families (Villegas-Morcillo et al., 2020). Notably, DL models, i.e., ToxDL and TOXIFY, incorporated end-to-end sequential learning to reduce dependency on handcrafted features and enabled adequate capture of latent toxicity outputs across diverse biological contexts (Villegas-Morcillo et al., 2020).

Multimodel architectural frameworks have integrated structural features, evolutionary profiles (PSSM), and protein LLM-based embeddings. ATSE demonstrated how attention mechanisms, graph-based learning, and transfer learning can enhance toxicity predictions by integrating both sequential and structural contexts (Wei et al., 2021). Notably, contemporary approaches have increasingly adopted hybrid and ensemble strategies, combining motif analysis, similarity searches, and ML predictions to reduce false positive outcomes by improving robustness (Sharma et al., 2022). Such integrative architectural frameworks have outperformed individual approaches by balancing specificity and sensitivity across diverse datasets (Sharma et al., 2022). Altogether, these are paradigm shifts toward multi-layered AI-driven predictive strategies, where toxicity must be embedded as core design constraints within AMP discovery pipelines.

#### 1.2.5 *In-silico* potency screening

One of the key limitations of early AMP models was that they performed binary classification, which was not sufficient for AMP translational prioritization. In practice, the key useful endpoints are quantitative efficacy outcomes, measured as MICs (lowest concentration of an antimicrobial agents which can inhibit visible growth of pathogens following incubation for a certain time period), and it favours triage, ranking, and optimization against specific drug-resistant pathogens (Wu et al., 2014; Chiu et al., 2026). In recent years, the computational approaches have shifted from sequence-based statistical screenings toward species-based MIC regressions, where the goals are to estimate how strongly novel AMPs inhibit bacterial activities against WHO priority pathogens (Wu et al., 2014; Chiu et al., 2026) (**Figure 9**; **Table 6**).

**Figure 9:**
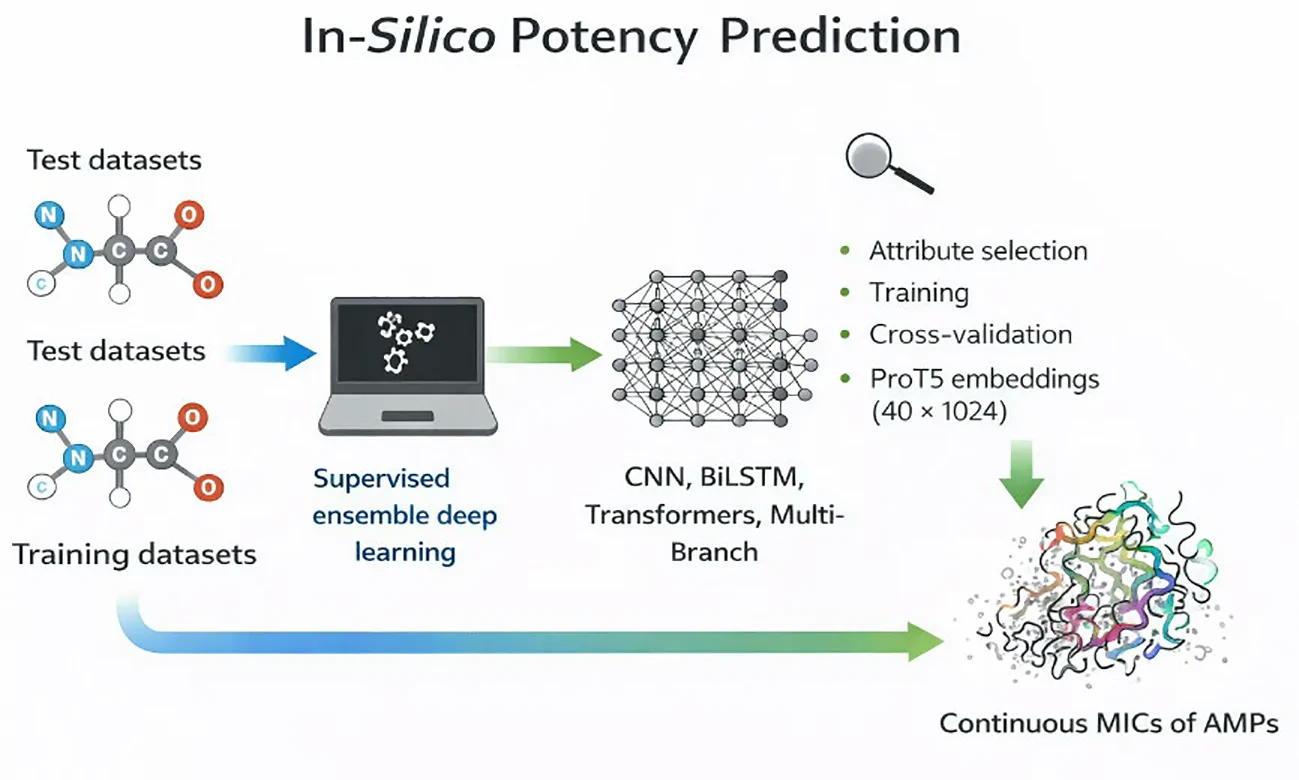
Supervised ML and DL-based potency-predicted NIC-regression models with integrated diverse architectural frameworks, i.e., adversarial learning & downstream scoring transformers, generators, and discriminators with regression heads for sequential representations, predict efficacy potentials. These models assign probability scores to reflect AMP likelihoods, enabling systematic ranking of high-confidence AMPs for downstream analysis. The image was created with BioRender (https://www.biorender.com).

Traditional potency-predicted models mainly relied on handcrafted sequential descriptors (Wu et al., 2014). An amino-acid activity contribution and linked substitution-based sequential optimization and wet-lab validation have demonstrated that computationally guided AMPs could achieve better potency both *in-vitro* and *in-vivo* (Wu et al., 2014). This has been followed by explicit MIC-prediction architectural frameworks, i.e., pseudo-amino-acid compositions alongside Gaussian kernel regression models for *E. coli*, establishing MIC predictions as distinctive computational tasks (Chiu et al., 2026). These early approaches demonstrated that antimicrobial potency outcomes were not only predictable but also empirically validated (Chiu et al., 2026).

Incorporation of DL regression architectural frameworks has improved predictive potency and accuracy (Dean et al., 2021). PepVAE coupled VAEs alongside activity-predicted models showed that gradient boosting performed better against *E. coli*; median *R*^2^ = 0.73 and root mean square error (RMSE)= 0.50. MBC-Attention approaches, combining multi-branch CNNs along with attention for *E. coli* MIC regression, demonstrated Pearson correlation = 0.775 and RMSE = 0.533, respectively (Dean et al., 2021). Recently, an ensemble model integrated genomic and sequence-derived features against WHO-priority pathogens (Pearson correlations of 0.756 for *S. aureus*, 0.781 for *E. coli*, and 0.802 for *P. aeruginosa*, respectively), marking the transition from generic activity screening towards pathogen-based quantitative potency predicted outcomes (Chung et al., 2024).

BERT-AmPEP60, fine-tuned ProtBERT for species-based MIC predictions and reported mean squared errors (MSEs) against *E. coli (*0.266) and *S. aureus* (0.303) and Pearson correlations of 0.796 and 0.753, respectively (Chung et al., 2024). *ESKAPEE*-MICpred incorporated two-input transfer-learning and ensemble architectural frameworks for pathogen-specific MIC predictions (Sharma et al., 2024). A recent pipeline (AMPredictor) has integrated generative designs alongside potency scorings: bifunctional AMP frameworks linked GAN generators to graph-convolutional MIC regressors, achieved RMSE = 0.5348 and Pearson= 0.7072, and validated 24 novel AMPs in an animal infection model (Dong et al., 2025b). Collectively, these architectural frameworks show how potency predictions have become a central layer within multi-purpose AMP pipelines to jointly optimize antimicrobial activity and novelty against resistant pathogens (Chung et al., 2024).

#### 1.2.6 *In-silico* novelty screening

*In silico* novelty screening must be considered one of the key components of the pipeline to limit the rediscovery of known AMPs. Novelty must be evaluated at two stages: first, against well-known peptide sequential spaces and proteins to limit homology; second, against genomics and transcriptomics spaces to determine whether the sequence maps back to untranslated regions, known genes and unannotated ORFs in pre-existing assemblies (Gertz et al., 2006; Camacho et al., 2009; Buchfink et al., 2021) (**Table 7**). In the past few years, it has relied on profile-based or alignment retrieval systems instead of traditional AI models (Gertz et al., 2006; Camacho et al., 2009). tBLASTn served as a standard translated-search-based method for mapping protein and peptide queries to nucleotide databases against all six reading frames (Gertz et al., 2006; Camacho et al., 2009) (**Figure 10**). tBLASTn has upgraded the reliability for nucleotide homologous searches by providing composition-oriented statistical outcomes, making it more effective for genome-level novelty outputs rather than naive translational searches (Gertz et al., 2006; Camacho et al., 2009) (**Figure 10**).

**Figure 10:**
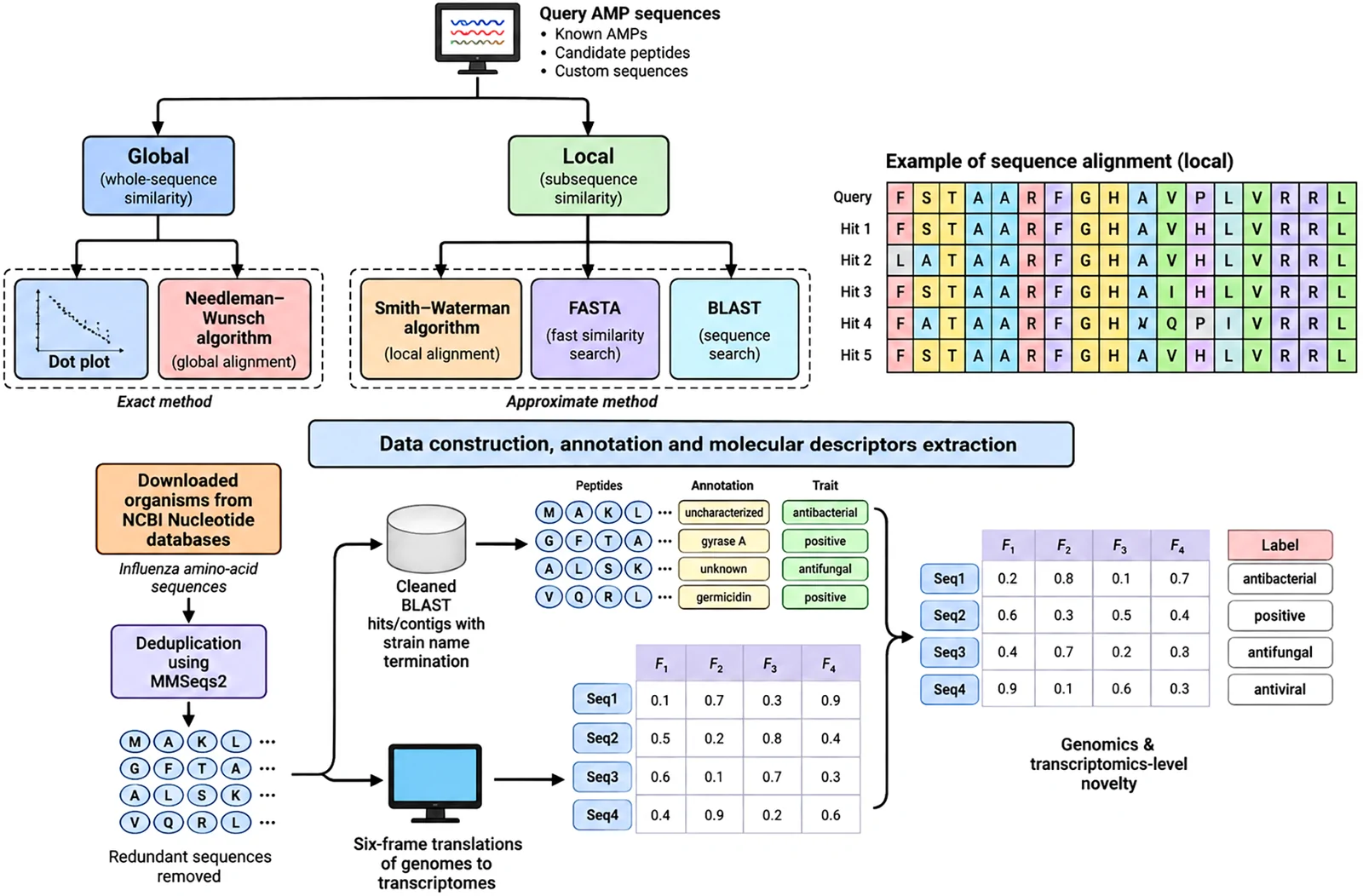
TBLASTN, using the NCBI core nucleotide database, to assess genomic-based novelty at the translated DNA level. Absence of significant hits indicated that AMPs did not correspond to known protein-coding genes, thereby providing genomics-based novelty confirmation, complementing the protein–based similarity screenings. The image was created with BioRender (https://www.biorender.com).

At the protein sequence level, novelty screening requires searching for candidates against commercially available large databases (Fu et al., 2012; Steinegger and Söding, 2017; Buchfink et al., 2021). MMseqs2 and DIAMOND have become crucial in recent times, as they conserve BLAST-like sensitivity and limit the time costs for large-scale datasets (Buchfink et al., 2021). DIAMOND has improved based-matched BLAST-like sensitivity and delivers approximately 80-fold speedup over BLASTP, as well as completing an nr versus UniRef50 tree-of-life scaling (Buchfink et al., 2021) (**Figure 11**). MMseqs2 demonstrated higher sensitivity than PSI-BLAST at far higher speeds; therefore, it can be run iteratively for larger workflows (Steinegger and Söding, 2017). To limit redundancy, CD-HIT considers practical upstream filters allowing it to quickly cluster highly similar sequences and limit inflated novelty due to near-duplicate inputs (Fu et al., 2012; Steinegger and Söding, 2017; Buchfink et al., 2021).

**Figure 11:**
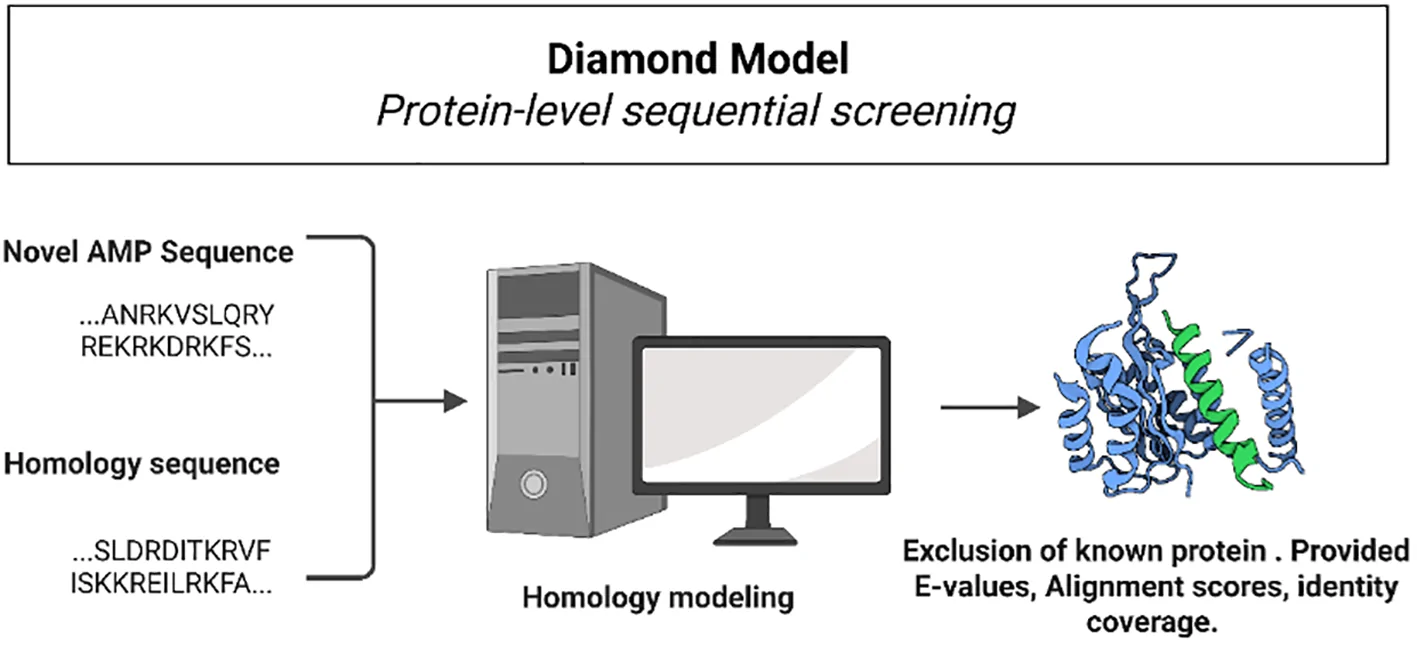
Diamond, a protein-based sequential similarity screening model, evaluated the AMPs’ novelty. Absence of significant alignments under strict identity parameters, i.e., E-values, alignment scores for retained peptides represented unreported sequences and classifying them as novel AMPs for downstream validations. The image was created with BioRender (https://www.biorender.com).

Pairwise local alignments alone can miss remote homologies for peptides that are short and compositionally biased towards precursor proteins (van Kempen et al., 2024). Many higher-confidence novelty workflows add domain-based or profile-HMM screenings. HMMER tools (jackhammer and phmmer) have provided profile-based sequential similarities that are considered more sensitive than simple pairwise methods for remote relationships (Finn et al., 2011). InterProScan has helped to determine whether a candidate is within an already known protein and motif architectural landscapes (Jones et al., 2014). Foldseek has extended novelty screenings beyond primary sequences by enabling fast structural searches; useful when sequential identities are lower and fold-based similarities suggest that candidates belong to pre-existing structural families rather than representing truly novel scaffolds (van Kempen et al., 2024).

For the AMP pipeline, multi-layer novelty screening is essential and requires removal of redundancy with CD-HITs. Peptides and proteins must be searched against well-known databases with BLASTP, MMseqs2 and DIAMOND, respectively, and promising AMPs must be backed onto genomic assemblies and transcript catalogues with tBLASTn to check mapping with known homologous regions (Fu et al., 2012; Steinegger and Söding, 2017; Buchfink et al., 2021). Later, InterProScan and HMMER can be applied for remote family assignments, and, when structural predictions are considered as an essential part of the workflows (Finn et al., 2011; Jones et al., 2014). Foldseek can be used to exclude structurally familiar scaffolds to limit rediscovery risks before downstream wet-lab validation (van Kempen et al., 2024).

#### 1.2.7 *In-silico* stability screening

Poor stability associated with rapid degradation by host and microbial proteases is one of the major translational barriers (Mathur et al., 2018). Therefore, *in-silico* AMP stability screening has become a valuable tool, with two key goals: firstly, to recognize protease-sensitive cleavage motifs that affect peptide half-life; secondly, to estimate circulation half-life as one of the proxies (Mathur et al., 2018) (**Figure 12**; **Table 8**). In recent times, advanced ML models incorporate evolutionary, sequential, structural, and physicochemical parameters, which are essential to identify early triage layers to flag unstable AMPs before synthesis and redesign to make protease resistance (Song et al., 2012; Mathur et al., 2018).

**Figure 12:**
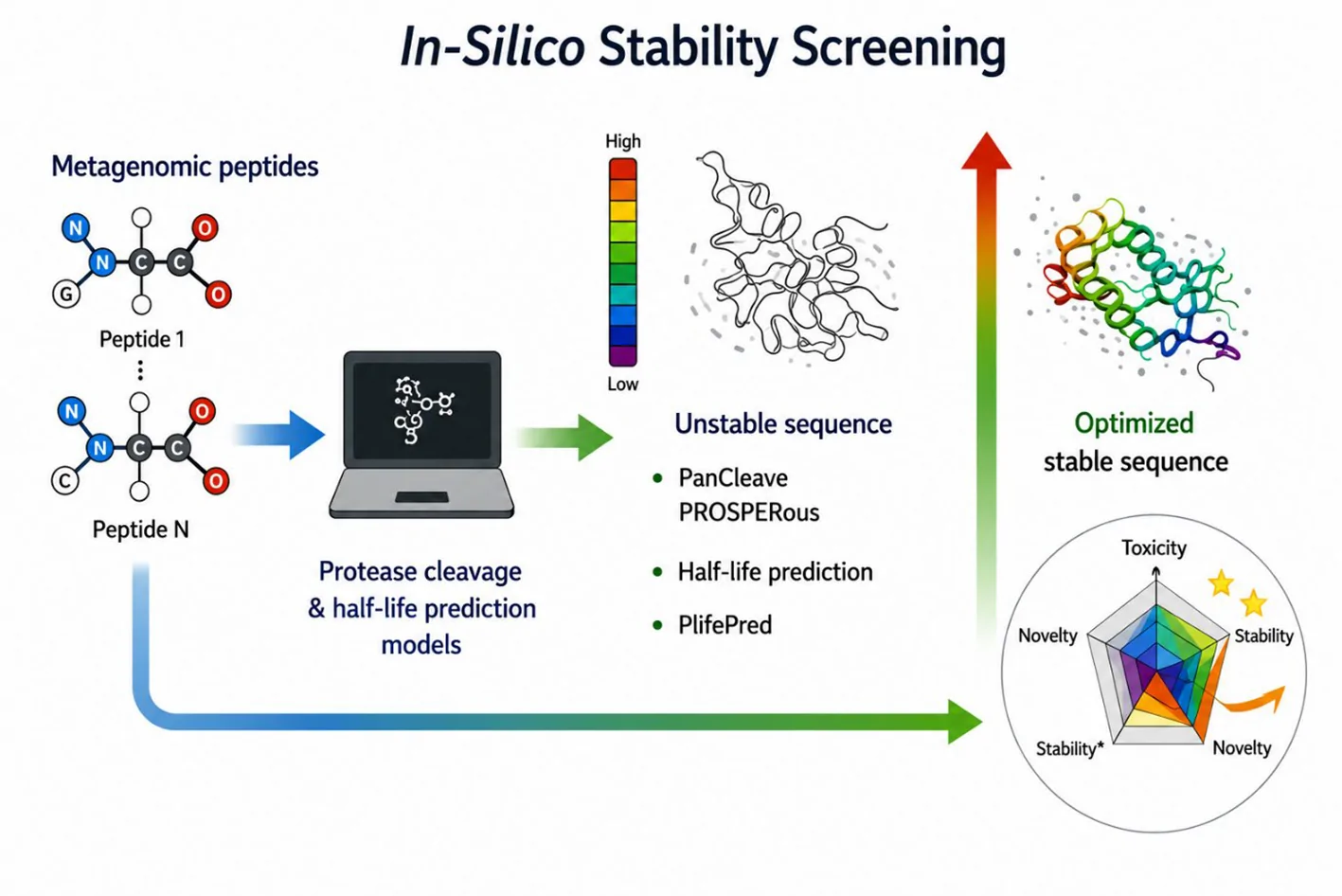
ML and DL-based models with integrated diverse architectural frameworks, i.e., adversarial learning & downstream scoring transformers, generators, and discriminators with regression heads for sequential representations, predict stability, enabling systematic ranking of high-confidence AMPs for downstream analysis. The image was created with BioRender (https://www.biorender.com).

PeptideCutter contains knowledge-based cleavage simulators and maps potential non-enzymatic and enzymatic cleavage sites, as well as providing rapid first-pass stability assessments by highlighting liability sites (Turalić et al., 2025). The protease-specific ML PROSPER can predict integrated feature-based cleavage sites across various proteases, and when followed by PROSPERous, can improve high-throughput predictions by expanding the coverage across 90 proteases (Song et al., 2012; Song et al., 2018). These tools are essential for AMP optimization, as shorter AMPs are sensitive to single cleavage events. Therefore, they provide advanced information regarding the peptide’s engineering, such as cyclization, residual substitutions, and D-amino-acid design strategies (Song et al., 2012; Song et al., 2018; Turalić et al., 2025).

Procleave has incorporated both sequential and 3D structural information to improve protease-based cleavage-site prediction, reflecting that local conformation often determines whether nominal motifs are actually cleaved (Li et al., 2020b). On the contrary, panCleave possesses pan-protease RF models for computational-based proteolysis and is used to mine encrypted AMPs from extinct human proteomes, leading to wet-lab validations of antimicrobial fragments, highlighting that cleavage predictions must be used constructively for AMP discovery (Kelley et al., 2024). PlifePred, a complementary cleavage-site tool, has addressed other stability dimensions by predicting peptide circulation half-life from experimentally validated data (Mathur et al., 2018). Altogether, these approaches highlight the need for adapting a multi-model workflow in which AMPs are screened for novelty, pharmacokinetic stability, potency, and toxicity before peptide synthesis.

#### 1.2.8 *In-silico* molecular docking approaches for structural optimization

Molecular docking (MD) is considered the principal component of structure-based AMP optimization, which predicts the binding modes and target specificity at atomic resolution. Unlike sequential-based predicted models, MD models ligand–receptor interactions to rationalise how AMPs interact with microbial membranes, enzymes, and intracellular targets (Davis and Baker, 2009; Lemmon and Meiler, 2012; DeLuca et al., 2015) (**Figure 13**; **Table 9**). In recent years, MD has advanced from rigid-body, physics-oriented scoring to hybrid architectural frameworks incorporating diverse ML, structural predictions, and flexible docking, improving performance metrics (Davis and Baker, 2009; Lemmon and Meiler, 2012; DeLuca et al., 2015). Traditional MD tools are widely used due to their interpretability and robustness, particularly during the initial stages of virtual screening (Morris et al., 2009) (**Figure 13**, **Table 9**).

**Figure 13:**
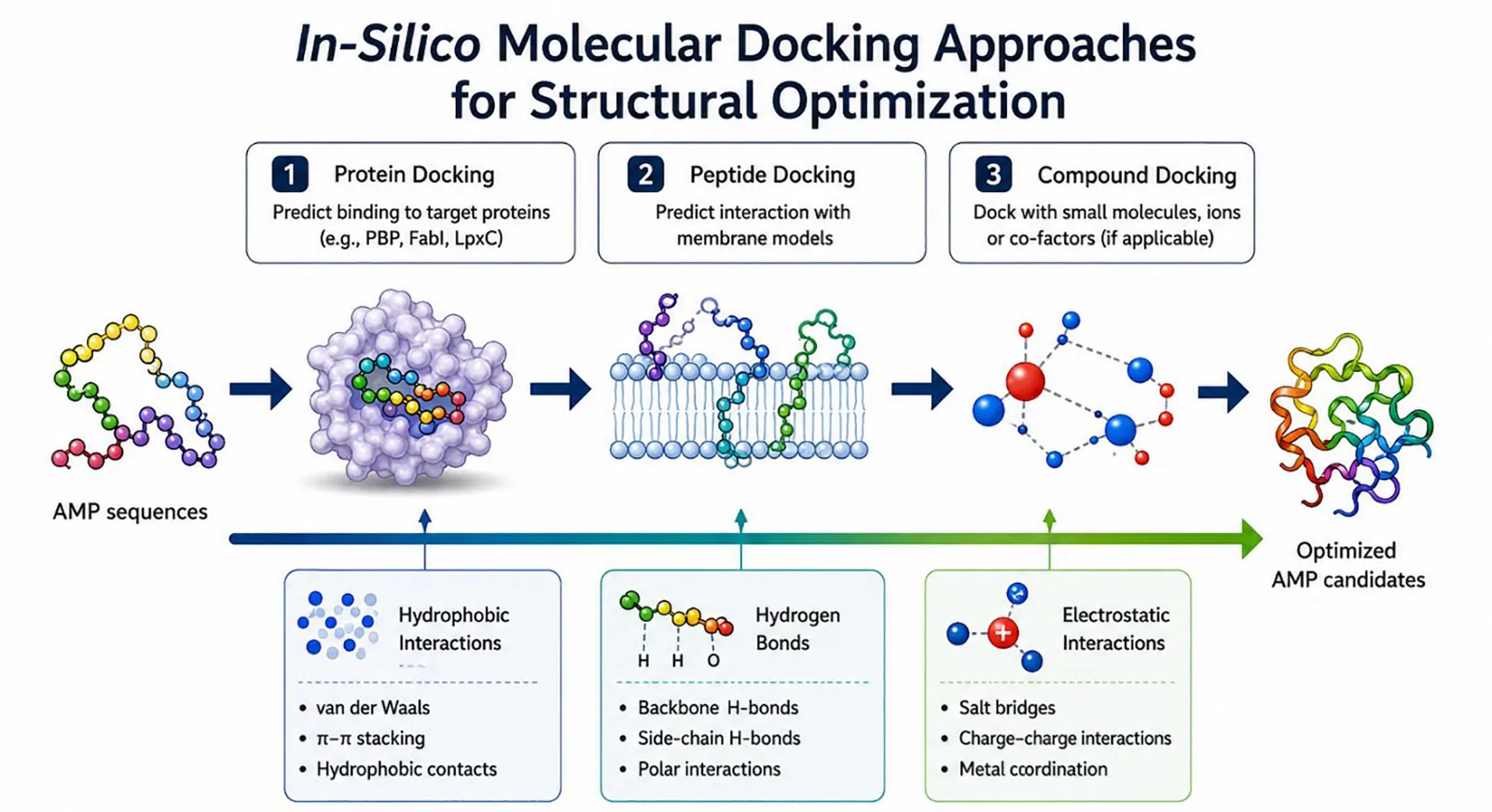
(1–3): AMP sequences are structurally modelled and subjected to docking against novel biological targets using protein-ligand and peptide-protein interactions. This enables the identification of key interaction determinants, such as hydrogen bonding, hydrophobic contacts, and electrostatic interactions, which collectively govern specificity and binding affinity. Structural refinements via iterative scoring and docking facilitate the optimization of peptide conformations, leading to enhanced functional activity. Integration of MD within multi-model pipeline strategies provides mechanistic bridges between sequence-based prediction and wet-lab validated AMP candidates. The image was created with BioRender (https://www.biorender.com).

AutoDock and AutoDock Vina have been foundational for peptide-protein docking. AutoDock Vina demonstrated better improvements in terms of speed and accuracy due to multi-threading and effective scoring functions, making them ideal for larger-scale virtual screening (Morris et al., 2009). Rosetta-based MD frameworks (RosettaLigand) have incorporated conformational sampling alongside physics-based energy functions to predict binding poses and optimized peptide-protein interactions, suitable for flexible ligands (Davis and Baker, 2009; Lemmon and Meiler, 2012; DeLuca et al., 2015). Notably, tools have extensively validated structure-based drug discovery and identified novel bioactive compounds. However, traditional MD approaches struggle with solvent effects, protein flexibility, and free-energy estimations (Davis and Baker, 2009; Lemmon and Meiler, 2012; DeLuca et al., 2015).

Recently, MD approaches have been transformed by integrating AI and structure-predicted models (Jumper et al., 2021; Abramson et al., 2024). AlphaFold has drastically increased the availability of high-quality protein structures, removing key bottlenecks for docking workflows (Jumper et al., 2021). AlphaFold3 can directly model protein-peptide and protein-ligand complexes, outperforming by predicting binding conformations (Abramson et al., 2024). Diffusion-based models (DiffDock) have reframed docking as a generative issue and significantly improved hit rates by correctly predicting bindings (Corso et al., 2022). Collectively, it highlights paradigm shifts toward data-oriented structural modelling by learning representations that complement and replace physics-based scoring.

MD approaches to identify novel bacterial targets against predicted AMPs require intensive computational resources. Latest MD frameworks support closed-loop optimization and larger-scale virtual screenings (Zhang et al., 2025; Voitsitskyi et al., 2026). Ultra-large-scale approaches screen billions of candidates against targeted structures, ensuring *in-silico* high-affinity binders and novel scaffold discovery before wet-lab validation (Zhang et al., 2025; Voitsitskyi et al., 2026). Hybrid systems have integrated docking with ML–based selection and scoring, e.g., LigDockTailor, to optimise performance outputs based on ligand properties (Zhang et al., 2025). Altogether, MD critically bridges sequence-oriented AMP design and structure-based optimization, enabling refinement for binding affinity and specificity within an advanced multi-model AMP pipeline.

## 2 Current AI-based approaches for mining, discovery, and optimization of AMPs

Evolution from classical ML to DL approaches and LLMs has revolutionized the computational toolkits for AMP mining and discovery in recent times (Fernandes et al., 2023; Perišić et al., 2025). However, increasing architectural frameworks and complexities do not guarantee better performance metrics (Fernandes et al., 2023; Perišić et al., 2025). There is no single AI framework that is optimal for AMP mining, identification, and characterization; therefore, it is dependent on the multitask models and available data resources (Fernandes et al., 2023; Perišić et al., 2025).

Multitask-matched models will be more beneficial than automatic progression based on their complex architectural frameworks. Classical models, RF, SVM, and XGBoost are effective for imbalanced and smaller datasets for rapid binary classification, while DL models, such as transformers, CNNs, and LSTMs, have outperformed for larger and labelled sequential datasets by learning long-range residual dependencies (Moretta et al., 2020; Musin and Asyanova, 2025; Yu et al., 2025). For structural tasks, 3D-aware geometric DL approaches are required for receptor-based designing and membrane-interactive modelling (Fernandes et al., 2023). Depending on the available computational resources, decisions regarding selecting various architectural frameworks vary with modality and predictive outcomes.

Models, such as AMPGen and EBAMP, have demonstrated that an architecture framework-based selection can be task-driven, i.e., RL-based and diffusion models were used for *de novo* AMP generation, while transformer-oriented MIC regressors predicted efficacy, and ensemble models favoured safety outcomes with multi-purpose screening when stability and interpretability were critical (Sharma et al., 2024; Veldsman et al., 2025; Zhao et al., 2025). Earlier evolutionary and genetic strategies demonstrated better multi-objective outputs regarding optimization, but their performances were hampered by the noisy biological data and limited datasets (Shin et al., 2022; Agüero-Chapin et al., 2023).

AMP workflows can span heterogeneous objectives, i.e., structure-based classification and designs, target-based specificity, MICs regression, and toxicity prediction (Wang et al., 2025; Wang et al., 2025d; Zhao et al., 2025). Multi-model evolution can expand the AMP discovery toolkit, depending on whether architectural choices are aligned with data quality and biological contexts, not complexity alone (Cao et al., 2023; Wang et al., 2025b). Therefore, a multi-model AI-driven pipeline strategy, as reviewed in the current paper, for predicting diverse architectural frameworks, aligning with the best practical implications for AMP discovery and optimization, is essential (Cao et al., 2023; Wang et al., 2025b) (**Figure 14**).

**Figure 14:**
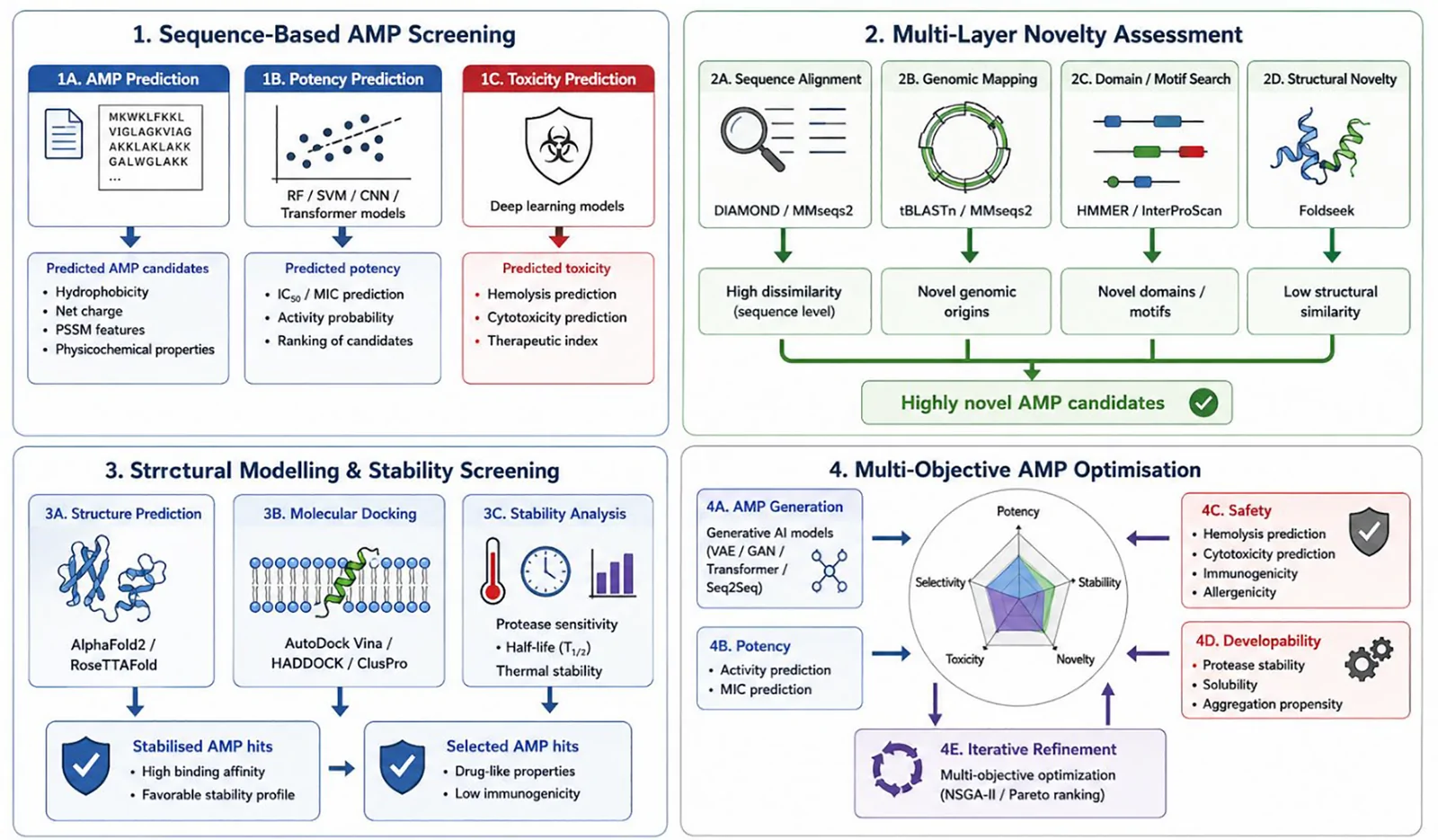
(1–4) The multi-model workflow presents a well-integrated in-silico pipeline strategy having sequence-based screening, novelty assessment, structural modelling, and multi-purpose optimization. (1A) AMPs are first identified using machine-learning- and deep–learning–based classifiers. Position-Specific Scoring Matrix (PSSM) descriptors capture evolutionary patterns of amino acid residues derived from sequence alignments to provide informative sequence signatures that enhance the accurate identification and classification of AMPs. (1B) Quantitative MIC regression models must be followed to prioritize sequences with better antimicrobial activity. (1C) Cytotoxicity and haemolysis prediction modules act as initial safety filters to exclude host-toxic candidates (Selvaraj et al., 2025). Novelty assessment combines sequence alignment, genomic mapping, and structural similarity analysis to ensure novel peptides (Sadeeq et al., 2025). Structural modelling using advanced protein-folding approaches alongside MD to evaluate peptide–target interactions and guide structure-based refinement. Stability screening assesses protease susceptibility (Palmo et al., 2026). All the components are integrated within iterative optimization frameworks to refine potency, toxicity, novelty, and stability, yielding high-confidence AMP candidates for downstream validation. The image was created with BioRender (https://www.biorender.com) & assisted by ChatGPT (GPT-5.5) for scientific visualization.

Multi-model AI-driven pipelines can facilitate mechanistic-informed routes to accelerate AMP discovery by progressing from wet-lab explorations to computation-based triage (Li et al., 2024; Wang et al., 2025d) (**Figure 14**). Therefore, mining larger metagenomic datasets using single-model screenings will be insufficient, as AMP discovery has intrinsic multi-objectives, i.e., predicted potency must be balanced against novelty, stability, bioactivity, and safety (**Figure 15**). Multi-model AI pipelines can address these concerns by combining foundation models to encode peptide grammar to enable targeted prioritization, discriminative predicted models for pathogen-specific potency, and safety filters to predict cytotoxicity and haemolysis (Li et al., 2024). Recent studies have explicitly demonstrated that multi-stage AI approaches have delivered validated AMPs against Gram-negative bacteria inside compressed design and test cycles, including pathogen-targeted outputs (Li et al., 2024; Wang et al., 2025d).

**Figure 15:**
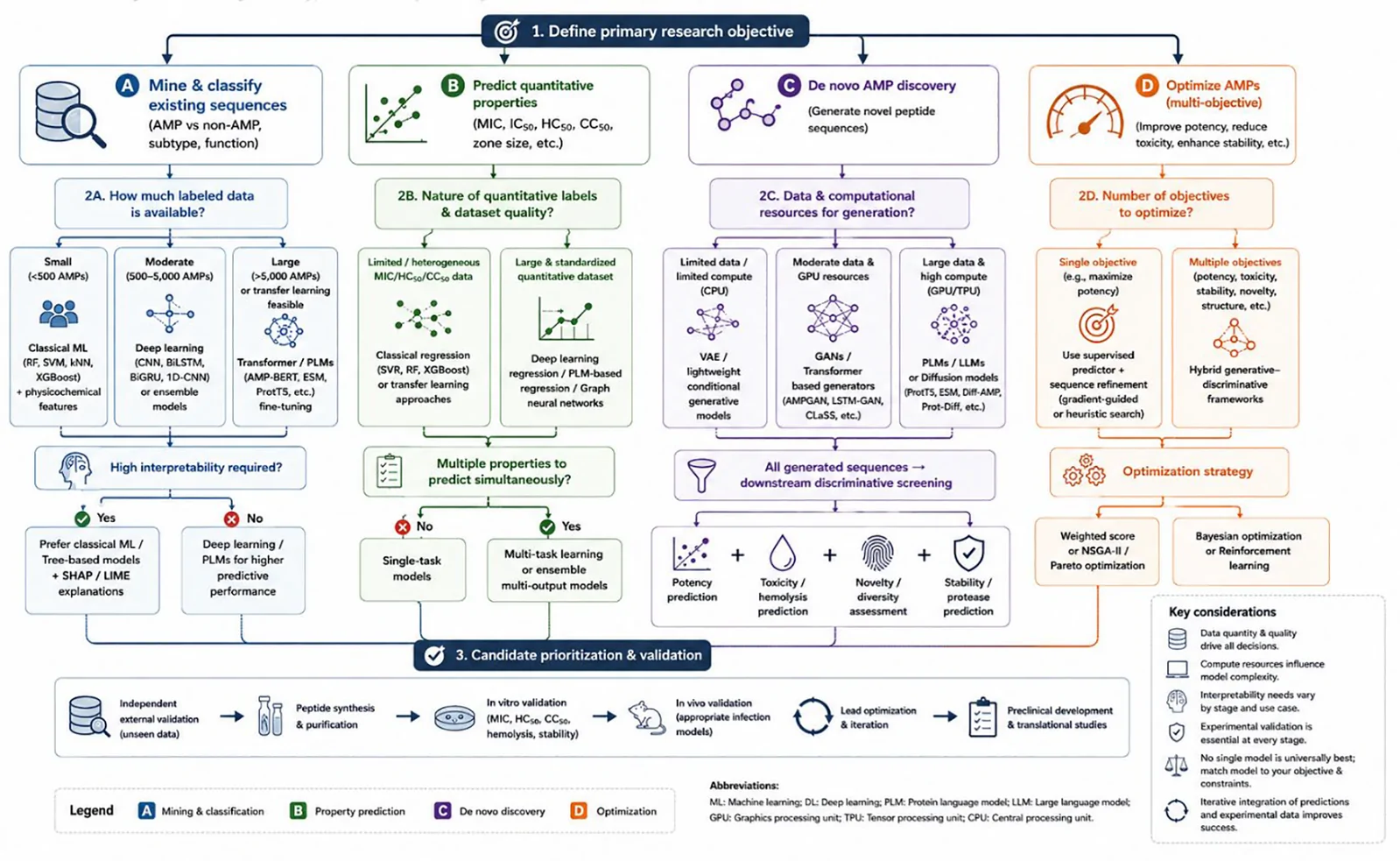
Decision tree for AI models selection for AMP discovery and optimization. **(a)** Classical or DL methods are selected based on labelled dataset size and interpretability requirements; **(b)** Multipurpose or regression models favour quantitative property prediction; **(c)** Generative models enable *de novo* AMP discovery; and **(d)** Hybrid frameworks enable multi-objective optimization. All pathways converge on AMP prioritization and independent experimental validation, emphasizing that model choice should be driven by data quality, resources and research objectives rather than architectural complexity alone. (https://www.biorender.com) & assisted by ChatGPT (GPT-5.5) for scientific visualization.

A multi-model AI-driven pipeline can mine and discover novel AMPs from diverse resources, i.e., natural, genomics, metagenomics, and *de novo* generative spaces, to predict efficacy and safety profile before wet-lab validations, thereby limiting the peptides that must be synthesized and assayed to achieve clinical relevance (Santos-Júnior et al., 2024; Li et al., 2025). It has been supported by recent ML and DL frameworks by identifying AMPs from microbiomes, subsequently *in-vitro* validation, and generating *de novo* AMPs with higher “hit rates” coupled with discriminative and safety models (Jin et al., 2025). MIC-based models against Gram-negative strains further prioritize WHO-listed pathogens to accelerate mechanism-driven selection (Bae et al., 2025).

Stronger computational and preclinical performances cannot guarantee downstream therapeutic successes. AMPGen provided contemporary examples of attrition at the computational-experimental interfaces (Jin et al., 2025). Although its XGBoost discriminators, diffusion-driven generators and LSTM-based target-specific scorer prioritized 40 *de novo* AMPs (Jin et al., 2025). 38 were synthesized and 31 of these met antimicrobial activity criteria (success rates of 81.58%) and were target-dependent: 19/20 synthesized AMPs targeting *E. coli* were active compared with only 8/18 targeting *S. aureus* (Jin et al., 2025). After multimodel computational filtering, a subset of highly prioritized AMPs had failed experiments, illustrating *in-silico outputs* alone cannot be considered as substitutes for biological validation (Jin et al., 2025).

At later translational stages, pexiganan (MSI-78) has provided a historical example (Lipsky et al., 2008). Despite having strong potency and advancement to phase III trials for managing infected diabetic foot ulcers, the clinical outcomes did not translate into regulatory approvals; the studies failed to meet their primary efficacy objectives (Lipsky et al., 2008). Iseganan, broad-spectrum protegrin analogues with better antimicrobial activity, failed to reduce ulcerative oral mucositis in a larger and randomized Phase III trial recruiting 545 patients (Trotti et al., 2004). Together, these studies have illustrated a continuum of attrition starting from computational predictions to wet-lab bioactivity, final clinical outcomes, and reinforce the urgent need for incorporating multimodel AMP pipelines to demonstrate pharmacological (PK/PD) and physiological constraints before clinical progression (Trotti et al., 2004; Lipsky et al., 2008). 

**Table 10** summarizes different multi-model ML approaches with reported wet-lab, *in-vitro*, and *in-vivo* validation by highlighting their predicted outcomes, essential to demonstrate translational impacts. Prioritizing multi-model AI approaches can offer real-world performance for antimicrobial outcomes, not just in-silico validity.

**Table 10:** Studies using multi-model hybrid-based frameworks for AMP predictions alongside experimental validation.

Multi-model AI	Architectural frameworks	Experimental-based evidence
a. Hybrid methods with ensemble frameworks		
AMPredictor	SVM, ESM-2, and MLP.	Predicted residual physicochemical descriptors using attention maps (Dong et al., 2025b; Sun, 2025).
AMP-BRET	RF and ProtBERT Transformers fine-tuned for regression.	Transfer learning and high precision from protein corpus to predict antimicrobial activity (Lee et al., 2023).
AMP-EF (AMP– Ensemble Framework)	Bi-LSTM, XGBoost, alongside attention layers.	Provided higher performance and generalization to predict antimicrobial activity (Zhang et al., 2023).
AMPpred-EL	ML ensemble with LightGBM and logistic Regression.	Combined various ML components to predict AMPs with antimicrobial activity (Lv et al., 2022).
AMPpred-DLFF	CNN and ESM-2-based feature extractions.	Synergized graphical attentions, convolutional features and protein-language embeddings for AMP bioactivity predictions (Chen et al., 2024).
AMP-META	LGBM with different AMP tools.	Predicted physicochemical descriptors and bioactivity using larger datasets (Jorge et al., 2012; Tsai et al., 2024).
AMPpredMFA	LSTM, CNN, Attention, & MLP.	Integrated CNN and LSTM to predict features (Li et al., 2024).
Ensemble MIC regression	Ensemble DL regression (multi-inputs).	MIC validation against *E. coli* and *P. aeruginosa* (Chung et al., 2024).
E-CLEAP	Ensemble of four MLP classifiers.	Ensemble neural classifiers for binary classification and antimicrobial activity predictions (Wang, 2024).
PepMultiFinder	Multi-filter ML approaches.	Predicted efficacy & safety (Duque-Salazar et al., 2020; Monsalve et al., 2024).
b. Hybrid protein language-based frameworks		
FusPB-ESM2	Neural networks with ProBERT and ESM-2 embeddings.	Fused two LLM embeddings for multi-purpose representations and microbial predictions (Wu et al., 2024; Wang et al., 2025a).
PGAT-ABPp	GAT with ProtT5 embeddings.	Integrated geometric DL for 3D structural representations to predict efficacy & safety profile (Hao et al., 2024).
c. Hybrid DL approaches based on deep representations & fusion features		
AFP-MFL	MLP with co-attention mechanistic approaches.	BLOSUM62 & ProtT5 predicted physicochemical properties, whereas MLP with co-attention showed multi-purpose fusion & antibacterial activity (Fang et al., 2023; Sultan et al., 2024).
AMPFinder	MLP, ProtTrans and OntoProtein.	Used protein sequences for functional annotations to predict pathogen-specific properties (Yang et al., 2023).
FSLSME	MLP and ESM-1.	Used different libraries for mining AMPs and to predict bioactivity (Szymczak et al., 2025).
Pang’s Approach	MLP with pre-trained BERT.	Predicted regression-oriented MICs and safety outputs (Pang et al., 2022).
UniproLcad	Bi-LSTM with attention methods, ESM-2, 1D-CNN, UniRep, ProtBERT.	Multi-purpose fusion models alongside competitive interpretability and accuracy (Wang et al., 2024).
UniDL4Biopep	CNNs with ESM-2 embeddings.	Pretrained model, with CNNs spatial features extracted from embeddings to predict antimicrobial & stability activity (Du et al., 2023).
d. Large language-based hybrid model		
AMP-Designer	LLM, foundation model with multi-purpose design components.	Validated AMPs’ bioactivity against Gram-negative bacteria, including *in-vivo* murine lung infection (Wang et al., 2025).
ProteoGPT	Pertained protein LLM (ProteoGPT) with sub-LLMs.	Validation against clinical Gram-negative superbugs (Wang et al., 2025d).
GPT-3-AMP	GPT-3 (generative) & SVM/RF (discriminative).	Uses GPT-3 to generate candidate peptides, using SVM/RF filters for antimicrobial activity (Wang et al., 2021; Li et al., 2025).
deepAMP	DL predictors alongside *in-silico* optimization (deepAMP-POM).	MIC testing against Gram-negative MDR pathogens (Li et al., 2024).
AMPGen	Diffusion-based generators, i.e., autoregressive diffusion alongside attention, XGBoost discriminator and LSTM scorer.	Target-specific, designed with *in-vitro* validation (Jin et al., 2025).
e. Hybrid models with CNNs, RNNs, or transformer-based frameworks		
AMPDeep	MLP with BERT, i.e., ProtBert.	Optimized bioavailability and safety profiles (Salem et al., 2022; Ramos-Llorens et al., 2024).
AI4AMP	Deep neural networks, i.e., CNNs, LSTM.	PC6 encodings to map sequences into physicochemical vectors to predict bioactivity (Lin et al., 2021).
AMPGen	STM, autoregressive diffusion models with XGBoost discriminators.	Predicted antimicrobial activity (Jin et al., 2025).
AMPTrans-LSTM	Transformers and LSTMs.	Protein databases to predict bioactivity (Mao et al., 2023).
sAMP-VGG16	VGG-based CNNs.	Optimized adaptive layers by incorporating LLB & deep CNNs for binary classification (Pandey and Srivastava, 2025; Pandey et al., 2025).
Capecchi et al.	Sequential GAN model, transformers & RNN classifiers.	Non-hemolytic AMPs targeting Gram-negative pathogens (Capecchi et al., 2021).
HydrAMP	cVAE-GAN hybrid.	Predicted efficacy and safety profiles (Szymczak et al., 2023).
LLAMP	Transformer-based protein LMs (ESM-2) for species-based potency.	MICs against Gram-negative and Gram-positive panels (Bae et al., 2025).
TACaPe-based (2025)	Transformer-based GAN models.	*In-vitro* MIC testing against *P. aeruginosa* (de Albernaz et al., 2025).

### 2.1 Current barriers and limitations to robust AI-driven AMP discovery

Despite rapid advances in ML, LLMs and generative AI, the predicted performance reported for AMP models must be interpreted cautiously. Major challenges arise from dataset bias and poor representation of biodiversity. Currently available AMP databases are enriched with previously characterized, cationic and relatively shorter membrane-active AMPs, whereas inactive AMP sequences have not been reported systematically (Torres et al., 2025; Veldsman et al., 2025). Mostly negative datasets are assembled computationally from different commercial resources, rather than experimentally validated AMPs (Torres et al., 2025). Such approaches can create differences and will allow models to adapt to dataset-specific characteristics rather than biologically relevant AMPs. Homologous AMPs and sequence redundancy distributed across test and training sets can cause information leakage, thereby overestimating the generalization (Wang, 2023; Wang et al., 2026). Therefore, future benchmarking requires similarity-aware train–test separations, redundancy-controlled partitions and evaluation against robust and independent sequence families and organism sources (Wang, 2023; Wang et al., 2026).

There is limited availability and heterogeneity of experimentally validated datasets, and only a smaller number of AMPs are characterized under universal experimental conditions. Activity annotations originating from heterogeneous assays may differ in microbial strains, medium compositions and endpoint definitions (Noor et al., 2025). MIC values obtained for the same AMPs may not be directly comparable across different studies. Moreover, safety endpoints are fragmented across cell lines, erythrocyte species and exposure conditions (Chen et al., 2025; Saleem et al., 2025). These concerns are particularly consequential for quantitative stability, haemolysis, potency, cytotoxicity, and pharmacokinetic models, where inconsistent experimental metadata can cause label noise (Chen et al., 2025; Saleem et al., 2025). Therefore, databases should evolve from sequence and bioactivity repositories towards assay-aware multimodel platforms having universal experimental protocols including pathogen-based and concentration-dependent information alongside serum binding, protease stability, and clinically-related safety endpoints (Chen et al., 2025; Saleem et al., 2025).

Model interpretability has represented an additional barrier to translational confidence. Different AI-oriented architectural frameworks can provide strong discriminative predictions with limited insights about sequence motifs and physicochemical interactions (Chen et al., 2025; Saleem et al., 2025). *Post hoc* approaches (e.g. attention mappings and residue-level saliency) can enhance interpretability, but do not propose biological mechanisms, particularly for generative models, where highly predicted AMP probabilities do not establish plausible mechanisms, microbial and resistance profiles (Musin and Asyanova, 2025). Therefore, explainable AI must be coupled with mechanistic assays and experimental perturbations, so computationally identified determinants can be tested independently.

Methodological transparency and reproducibility have remained inconsistent across AMP modelling studies; differences in databases, redundancy thresholds, sequential preprocessing, hyperparameters, random seeds and data splitting can produce variations amongst comparable methods (Mittal et al., 2025). In some studies, training sequences may occur accidentally within benchmark sets, while unavailable source code and pretrained weights can limit independent reproduction (Chen et al., 2025; Saleem et al., 2025).

Absence of universally accepted benchmarking protocols for AMP classifiers, and differences within the benchmark compositions have made direct comparison problematic. This is amplified by class imbalances, where higher AUROC may provide incomplete performance descriptions (Mittal et al., 2025). Generative models can be assessed using divergent measures, such as validity, novelty and predicted activity, while quantitative MIC models can be evaluated using heterogeneous transformations, regression metrics and pathogen subsets (Noor et al., 2025). Temporal validations where models can be trained on available data, subsequently tested on reported AMPs can provide especially rigorous measures of prospective performance (Sun, 2025).

Computational performance cannot equate with therapeutic performance. Most AI-generated AMPs have been evaluated *in-silico*, and only smaller proportions have progressed through synthesis, serum stability assessments and *in-vivo* validations (MIC determination and cell toxicity) (Chen et al., 2025; Saleem et al., 2025). Physiological factors (ionic strength, serum and protein binding, proteolysis, aggregation, biodistribution and immune interactions) can substantially alter activities (Chen et al., 2025; Saleem et al., 2025). Multimodel pipelines must be viewed as prioritization platforms for the next phases of AI-driven AMP discovery, requiring prospective design-build and test-learn cycles, where standardized experiments will update computational models, essential for moving from retrospective predictions towards clinically-oriented AMP engineering.

### 2.2 Future directives

Convergence of advances within structural biology, sequential modelling, and high-throughput computational approaches has favoured a shift from fragmented predicted workflows toward a well-integrated and multi-purpose strategy for AMP discovery (Palmo et al., 2026). Whilst traditional linear approaches evaluate stability, activity and toxicity sequentially, the proposed methodologies adapt closed-loop, multi-model architectural frameworks, where AMPs are iteratively refined across orthogonal predictive multi-layers (Wang et al., 2025). This advanced AI-driven design has enabled simultaneous optimization of structural and proteolytic compatibility, novelty, potency, and host safety, thereby addressing the key bottlenecks historically limited to AMPs’ clinical translation (Saleem et al., 2026).

Multi-model AI-driven exhibits a hierarchical screening strategy. Candidate sequences can be derived from metagenomics or curated libraries and are evaluated using discriminative AMP classifiers, which later pass through quantitative MIC regression frameworks to predict and prioritize AMPs against resistant pathogens (Guan et al., 2025). Parallelly, hemolytic and cytotoxicity models serve as initial safety filters to exclude AMPs predicted to have higher cytotoxicity (Xie et al., 2025). Early-stage incorporation of potency and safety filters demonstrates key departures from traditional drug discovery pipelines, where cytotoxicity is evaluated following wet-lab validation (Xie et al., 2025).

Following efficacy and safety filtering, shortlisted AMPs must undergo novelty assessments; genomic search and profile-based homology detection must translate with rapid sequential alignment tools (DIAMOND, MMseqs2) to identify peptides that are active and distinctive from known AMPs and conserved protein domains (Steinegger and Söding, 2017; Buchfink et al., 2021). Later, they must be structurally characterized using sophisticated structure prediction models (AlphaFold2/3) and downstream molecular interactions against novel targets, providing insights into binding orientations and potential mechanistic approaches facilitating rational optimization (Jumper et al., 2021; Voitsitskyi et al., 2026).

Integration of degradation-aware and stability tools must be used to predict proteolytic susceptibility and AMP half-life to exclude unstable candidates (Song et al., 2012; Li et al., 2020b). Peptide modifications, such as cyclization, residual substitution, and incorporation of non-natural components, can enhance stability without impairing bactericidal activity, enabling iterative closed-loop optimization cycles to improve diverse objectives simultaneously (Song et al., 2018).

At present, the absence of standardized datasets demonstrating the serum protein binding effects on antimicrobial activity is one of the major obstacles associated with AI-driven drug discovery (Wang, 2023; Wang et al., 2026). Most AMPs possess better potency outcomes under laboratory conditions, but exhibit reduced efficacy within physiological environments due to non-specific interactions with circulating serum proteins, i.e., albumin, globulin, and lipoproteins (Wang, 2023; Wang et al., 2026).

There is a need to develop larger-scale and wet-lab-validated serum-binding databases for systematically quantifying AMP in the presence of human serum. Therefore, integration of serum-binding data resources alongside ML, DL and generative AI frameworks would enable the identification, prediction and optimization of AMPs’ performance under biologically relevant conditions, thereby improving the clinical translation of next-generation AMP from bench to bedside (Wang, 2023; Wang et al., 2026).

Despite rapid advancements in AI-assisted AMP discovery, clinical translation depends on establishing regulatory and manufacturing frameworks (Zhao et al., 2026). Regulatory assessments require demonstration of higher predictive accuracies; therefore, developers must provide documentation of model provenance, predefined decision thresholds and to what extent computational predictions will contribute towards AMP selection and sequence modification (Zhao et al., 2026). Additional scrutiny is required when models can introduce unusual sequence motifs and chemical modifications for which pharmacological evidence is limited. It must converge with conventional chemistry, control and manufacturing requirements (Zhao et al., 2026).

At present, manufacturing has become challenging for highly modified, cyclized and non-canonical AMPs, where solid-phase synthesis, analytical characterization and purification have increased production cost and complicated scale-up (Chen et al., 2025; Saleem et al., 2025). Future pipelines must incorporate developability and manufacturability as one of explicit design objectives, so that AMPs characterized and optimized using computational screenings will be compatible with scalable manufacturing production and subsequent regulatory evaluation (Zhang et al., 2025).

From retrospective validation to larger prospective experimental studies, AI-designed AMPs must be evaluated against several clinical and resistant strains using standardized *in-vitro* assays. Clinical translation must demonstrate across genetically and phenotypically diverse pathogens, followed by incorporation of protein-binding and protease stability, cytotoxicity, and pharmacodynamic–pharmacokinetic relationships, before advancing towards animal models (Su et al., 2025). External validations across different laboratories will be mandatory to determine model performance beyond the datasets (Zhao et al., 2026). Most credible pipelines must route towards clinical applications possessing prospective design-build and test-learn frameworks, where AI-predicted AMPs must be subjected to diverse experimental filters and the resulting biological data must be returned to the computational pipelines. Such iterative validations will enable AMP discovery by progressing from high-performing *in-silico* drug design towards reproducible AMPs with better therapeutic potential (Torres et al., 2025).

### 2.3 Conclusion

An integrated, multi-model, advanced AI-driven pipeline demonstrates transitions toward systematic AMP design and optimization, where different predicted modalities operate in a concerted manner rather than in isolation. Therefore, by embedding functional, structural, and pharmacological constraints within a unified and well-integrated framework, this approach favours effective navigation of peptide design spacing to reduce later-stage attrition. These approaches are well-suited for high-throughput computing, where larger-scale AMP mining, generation and evaluation can be performed in parallel to support rapid translation from *in-silico* discovery to wet-lab validation for clinical translation. This multi-model strategy provides generalizable blueprints for developing next-generation antimicrobial agents to combat the global AMR crisis in the era of computational biology.
